# Edge stabilization in reduced-dimensional perovskites

**DOI:** 10.1038/s41467-019-13944-2

**Published:** 2020-01-10

**Authors:** Li Na Quan, Dongxin Ma, Yongbiao Zhao, Oleksandr Voznyy, Haifeng Yuan, Eva Bladt, Jun Pan, F. Pelayo García de Arquer, Randy Sabatini, Zachary Piontkowski, Abdul-Hamid Emwas, Petar Todorović, Rafael Quintero-Bermudez, Grant Walters, James Z. Fan, Mengxia Liu, Hairen Tan, Makhsud I. Saidaminov, Liang Gao, Yiying Li, Dalaver H. Anjum, Nini Wei, Jiang Tang, David W. McCamant, Maarten B. J. Roeffaers, Sara Bals, Johan Hofkens, Osman M. Bakr, Zheng-Hong Lu, Edward H. Sargent

**Affiliations:** 1 Department of Electrical and Computer Engineering, University of Toronto, 10 King’s College Road, Toronto, ON M5S 3G4 Canada; 2 Department of Materials Science and Engineering, University of Toronto, 184 College Street, Toronto, ON M5S 3E4 Canada; 3 Department of Chemistry, KU Leuven, Celestijnenlaan 200F, B-3001 Leuven, Belgium; 4 EMAT, University of Antwerp, Groenenborgerlaan 171, 2020 Antwerp, Belgium; 5 Division of Physical Science and Engineering, King Abdullah University of Science and Technology (KAUST), Thuwal, 23955-6900 Saudi Arabia; 6 Department of Chemistry, University of Rochester, 120 Trustee Rd., Rochester, NY NY14627 USA; 7 Core Labs, King Abdullah University of Science and Technology, Thuwal, 23955-6900 Saudi Arabia; 8 Wuhan National Laboratory for Optoelectronics (WNLO), Huazhong University of Science and Technology (HUST), 430074 Wuhan, China; 9 Centre for Surface Chemistry and Catalysis, KU Leuven, Celestijnenlaan 200F, B-3001 Leuven, Belgium; 10 Max Planck Institute for Polymer Research, Ackermannweg 10, Mainz, 55128 Germany; 11 Present Address: College of Materials of Science and Engineering, Zhejiang University of Technology, Hangzhou, China

**Keywords:** Engineering, Materials science

## Abstract

Reduced-dimensional perovskites are attractive light-emitting materials due to their efficient luminescence, color purity, tunable bandgap, and structural diversity. A major limitation in perovskite light-emitting diodes is their limited operational stability. Here we demonstrate that rapid photodegradation arises from edge-initiated photooxidation, wherein oxidative attack is powered by photogenerated and electrically-injected carriers that diffuse to the nanoplatelet edges and produce superoxide. We report an edge-stabilization strategy wherein phosphine oxides passivate unsaturated lead sites during perovskite crystallization. With this approach, we synthesize reduced-dimensional perovskites that exhibit 97 ± 3% photoluminescence quantum yields and stabilities that exceed 300 h upon continuous illumination in an air ambient. We achieve green-emitting devices with a peak external quantum efficiency (EQE) of 14% at 1000 cd m^−2^; their maximum luminance is 4.5 × 10^4^ cd m^−2^ (corresponding to an EQE of 5%); and, at 4000 cd m^−2^, they achieve an operational half-lifetime of 3.5 h.

## Abstract (web summary)

Reduced-dimensional halide perovskites are promising for light-emitting diodes but suffer from photo-degradation. Here Quan et al. identify the edge of the perovskite nanoplatelets as the degradation channels and use phosphine oxides to passivate the edges and boost device performance and lifetime.

## Introduction

Reduced-dimensional metal halide perovskites (MHPs) are an emerging class of materials that hold advantages in optoelectronics relative to conventional three-dimensional (3D) MHPs^1–7^. Reduced-dimensional MHPs are intermediate between 3D and two-dimensional (2D) perovskites: they are synthesized via control over the concentration of large organic cations incorporated in between perovskite layers. The added organic cations confining perovskite layers increase the formation energy and mitigate chemical degradation in the presence of moisture^8,9^, enabling solar cells exhibiting improved stability compared to their 3D counterparts^10–12^. Strong and tunable confinement allows the exciton binding energy to be increased well above the thermal dissociation threshold, enabling increased radiative rates for light-emission applications^13–16^. The multiple quantum wells of varying thicknesses provide cascade energy transfer among domains with different bandgaps, leading to photoluminescence quantum yields (PLQYs) of over 60% at low pump power densities^17^.

However, reduced-dimensional MHPs still show limited stability under sustained photoexcitation and electrical injection, and this remains a roadblock to their deployment in light-emitting diodes (LEDs)^17,18^. Understanding of the mechanisms behind this degradation have benefited from a number of important studies: it was shown that long-lived free carriers accumulate at the edge of reduced-dimensional MHPs, leading to a high density of dangling bonds and unsaturated atoms. The edge states in reduced-dimensional MHPs refer to the states that are chemically unstable, structurally uncovered by organic amines. These exciton-accepting edge states are susceptible to moisture and oxygen, and under photoexcitation they are the recipients of significant carrier transfer, especially in wide-bandgap materials^19^.

Here we investigate the degradation mechanism in reduced-dimensional MHPs using a combined computational and experimental strategy. We study the role of these sites in photodegradation and then devise an edge-stabilization strategy to mitigate this problem. This enables us to report the longest device operational lifetime at high luminance (4000 cd m^−2^), by a margin of >21 times, relative to the best prior report (at the initial luminance of 3800 cd m^−2^, with *T*_50_ = 10 min)^20^.

## Results

### Structural analysis of reduced-dimensional perovskites

We focused on reduced-dimensional MHPs with a stoichiometry of PEA_2_Cs_2.4_MA_0.6_Pb_4_Br_13_ (here PEA is phenylethylammonium and MA is methylammonium). We synthesized the perovskites using a one-step spin-coating method. The films showed green emission peaked at 517 nm and exhibited a high PLQY of 60%. The optimization of the Cs-to-MA ratio revealed that an appropriate amount of MA was important to achieve high PLQYs (Supplementary Table 1)^18^.

**Fig. 1:**
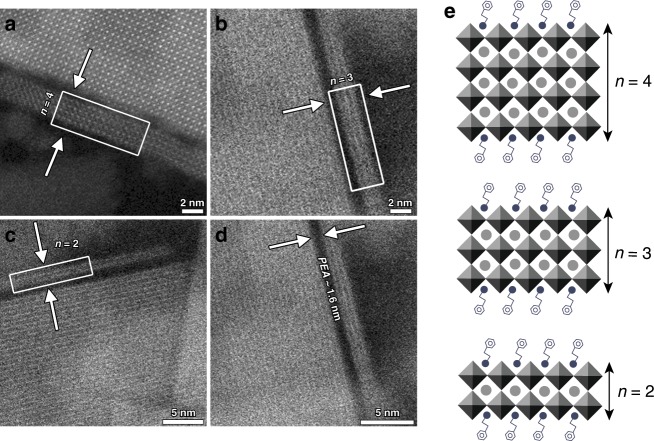
Visualization of reduced-dimensional perovskites. High-angle annular dark field (HAADF) scanning transmission electron microscopy (STEM) images of the layered perovskites exhibiting domains with different number of layers (**a**–**d**), where in **a**, a four-layered structure was observed.

We obtained the nanoplatelet thickness distribution required for energy funneling. We used aberration-corrected low-dose high-angle annular dark field (HAADF) scanning transmission electron microscopy (STEM) (Fig. 1 and Supplementary Fig. 1). Individual sheets consisting of two to four PbBr_6_ unit cells were clearly resolved. The distance between stacked sheets was 1.5–1.6 nm, corresponding to the PEA organic interlayer thickness. Multiple step edges were also resolved in STEM images (Supplementary Fig. 2), but could not exclude that such a step would be induced by the interaction of the highly energetic electrons with the perovskite outer surface.

### Conceptual design of edge-stabilization strategy

Previous work on 3D perovskite solar cells^21^ has shown that, when MHPs are photoexcited, the surface-localized excitons or carriers transfer to the adsorbed oxygen molecules, turning them into superoxide (O_2_^−^) that triggers perovskite oxidation and decomposition. A photodegradation pathway is triggered when an electron is transferred from the perovskite to O_2_, creating a superoxide (O_2_^−^) that irreversibly splits and converts into a chemisorbed oxide species.

**Fig. 2:**
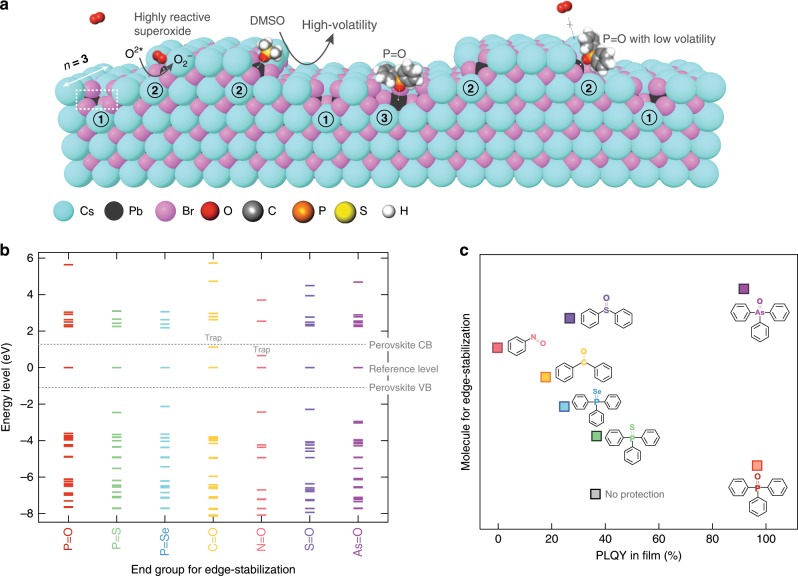
Photo-induced degradation mechanisms and edge-stabilization strategy. **a** Schematic illustrating imperfect edges in *n* = 3 reduced-dimensional perovskites of PEA_2_Cs_*n*−1_Pb_*n*_Br_3*n*+1_ and proposed reaction pathway of superoxide production under photoexcitation, including (1) missing Cs^+^ or PEA^+^ at the edge sites, (2) missing Cs^+^ or PEA^+^ at the corner sites, (3) desorbed CsBr or PEABr near the defect. **b** Energy level alignment obtained from DFT calculations. **c** PLQYs of the perovskites treated using different molecules.

Density functional theory (DFT) calculations indicated that the unsaturated Pb dangling bonds do not, on their own, form trap states (the unsaturated Pb dangling bonds were exposed due to the loss of PEA^+^ capping ligand or PEABr) (Fig. 2a and Supplementary Figs. 3–7)^22–24^. However, they remain susceptible to the adsorption of a variety of nucleophilic molecules (e.g. oxygen molecules) that readily forms a dative bond with the surface. The physically adsorbed oxygen molecules result in electronic traps in the perovskite bandgap, a phenomenon also seen in other semiconductors (Supplementary Fig. 7)^25,26^.

We hypothesized that introducing a benign Lewis base adduct—one that outcompeted oxygen adsorption—could improve the stability of perovskites in an oxygen-rich environment. Typical Lewis base polar aprotic solvents have been applied to dissolve perovskite precursors, such as dimethyl sulfoxide (DMSO), dimethyl formamide (DMF) and *N*-methyl-2-pyrrolidone (NMP), or form adducts with the metal halides, and therefore are widely used to impede the fast formation of perovskite crystals and to control film morphology^27–29^. However, these Lewis base–metal complexes formed with volatile solvents failed to withstand the annealing step during film fabrication (Fig. 2a). Reliance on this approach therefore left metal dangling bonds exposed to oxygen attack^30^.

We sought the materials that would combine the desired electronic and edge-stabilizing properties, and that would be sufficiently robust to remain following annealing (Fig. 2b). We tested various organic compounds both computationally and experimentally. We first performed DFT simulations to calculate the binding energy and investigate the energy level alignments by using a hybrid exchange-correlation functional of B3LYP (Methods). We started from organic molecules with a P=X end group (X is oxygen, sulfur or selenium), such as triphenylphosphine oxide (TPPO), triphenylphosphine sulfide (TPPS) and triphenylphosphine selenide (TPPSe). We found that the P=O:Pb dative bond showed a strongest binding energy of 1.1 eV. We also explored other oxides with a Y=O end group (Y is carbon, nitrogen, sulfur or arsenic), such as nitrosobenzene (PNO), benzophenone (DPCO), diphenyl sulfoxide (DPSO) or triphenylarsine oxide (TPAsO) to compare with TPPO. We found that the P=O:Pb dative bond was also stronger than S=O:Pb (0.8 eV) and Pb binding with the physically absorbed O_2_ (0.3 eV). Energy level alignment calculation revealed that PNO and DPCO introduce states that reside within the perovskite bandgap (Fig. 2b). These trends were seen in PLQY studies, which indicated that perovskites edge-stabilized by TPPO and TPAsO showed superior PLQYs of 97% and 92%, respectively, much higher than films treated with other molecules (with PLQYs from 0.1% to 40%) (Fig. 2c).

### Photothermal stability

**Fig. 3:**
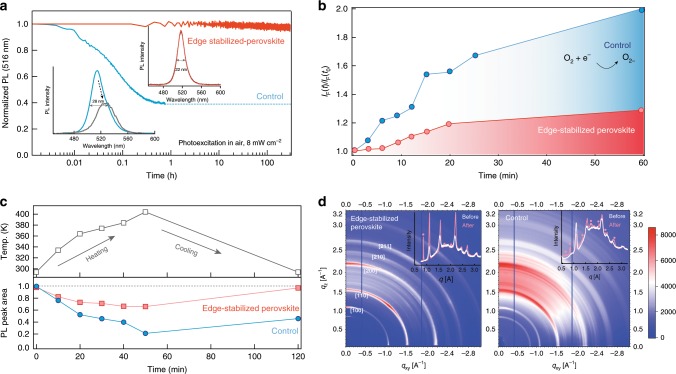
Photothermal stability. **a** PL stability under continuous excitation under a 374-nm laser diode. The inset shows PL spectra of the untreated control and edge-stabilized sample before (in red and blue, respectively) and after (in gray) measurement. **b** Normalized fluorescence intensity of the superoxide probe solution. **c** Thermal stability of the untreated control and edge-stabilized perovskite under a continuous heat stress. **d** In situ GIWAXS of the untreated control and edge-stabilized perovskite. The films were gradually annealed up to 150 °C, let there for 30 min before cooling down and measured. The inset curves show the out-of-plane line profiles before and after heat stress.

Photoluminescence (PL) spectra of edge-stabilized perovskite films show narrower linewidth compare with control perovskites (Supplementary Fig. 8). The phosphine oxides introduced in situ during the perovskite crystallization process, removed edge state defects and also tightened the distribution of quantum wells, resulting in a narrowband emission and enabling fast energy funneling in cascade energy structure. We then monitored the PL stability of the TPPO-treated perovskites and untreated controls under continuous excitation by using a 374-nm laser diode (8 mW cm^−2^) in air with a relative humidity of 10% (Fig. 3a). The emission of the untreated controls degraded to 40% of its initial value within 1 h, and was accompanied by broadened and red-shifted spectra. By contrast, the TPPO-treated perovskites retained their initial brightness and emission peak position over the course of continuous illumination for 300 h. To ascertain whether superoxide production played a key role in these films under photoexcitation, we measured superoxide generation under illumination. We used a superoxide-sensitive dye as a reporter (Supplementary Fig. 9), placed within the films, and monitored the increase of PL intensity of the dye (peaked at 610 nm) associated with the evolution of superoxide (Fig. 3b). The PL intensity of the dye doubled within 1 h relative to the initial intensity. Here we studied the ratio of *I*_F_(*t*) / *I*_F_(*t*_0_), where *I*_F_(*t*) shows the fluorescence intensity at time *t* and *I*_F_(*t*_0_) indicates the background fluorescence intensity of the probe the superoxide probe dye molecule at *t* = 0. This figure reports the yield of superoxide generation, and the result agrees well with the prior reports with bulk perovskites degradation mechanisms. The emissive properties of the edge-stabilized perovskites exhibited reversibility following thermal stress, and recovered their near-unity PLQY following heating at 150 °C (Fig. 3c and Supplementary Fig. 10). In the case of the untreated control, most of the PL intensity was lost during the heating process, and only 50% was recovered after cooling to room temperature. In addition, in situ grazing incidence wide-angle X-ray scattering (GIWAXS) (Fig. 3d and Supplementary Fig. 11) showed that edge-stabilized perovskites kept their initial structural phase and crystallinity following heat stress. In contrast, the untreated control exhibited increased disorder, as evidenced by broader rings, and the appearance of additional peaks associates with structural degradation.

### Edge-stabilization mechanism

**Fig. 4:**
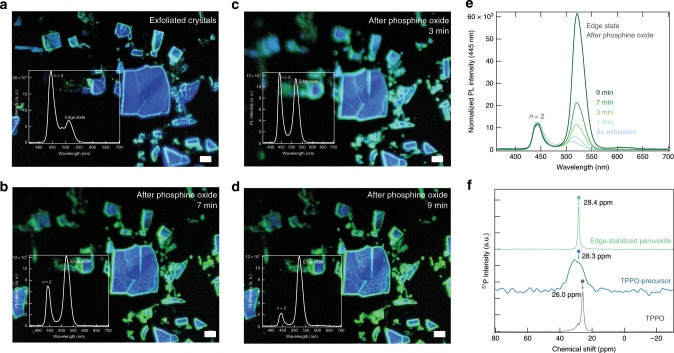
Origins of edge stabilization. **a** Microscopic image of the mechanically exfoliated PEA_2_CsPb_2_Br_7_ (*n* *=* 2) single crystal under continuous excitation by using a 374-nm laser diode. The inset shows PL spectra, from the both intrinsic (*λ*_em_ = 450 nm) and edge state (*λ*_em_ = 510 nm) emission. Scale bar is 10 μm. **b–d** Microscopic images as function of time after in situ addition of TPPO solution. **e** Normalized PL spectra extracted from the microscope images. Scale bar is 10 μm. **f** ^31^P-NMR spectra of TPPO only, TPPO-precursor (TPPO-PbBr_2_) and TPPO-perovskites to monitor the interaction between P=O and Pb in perovskites.

Next we fabricated single crystals of the reduced-dimensional MHPs with the composition of PEA_2_CsPb_2_Br_7_ (Supplementary Fig. 12) and exfoliated them into few-hundred-micrometer-sized thin flakes to distinguish the edge and the center of the samples using optical microscopy. Figure 4a–e shows the mechanically exfoliated PEA_2_CsPb_2_Br_7_ (*n* = 2) crystals, which have been reported to exhibit edge states with PL emission from low energy (520 nm) when exposed to air^31^. This is assigned to the stochastic loss of PEA and formation of bulk CsPbBr_3_ perovskites. The PL intensity from edge states increased twofold upon in situ addition of phosphine oxide molecules. We attribute this to the passivation of bulk perovskites located at the crystal edges. The PL from the bulk crystal (*n* = 2) did not change an observation we account for by noting that these crystals were oriented along the <001> direction, and the organic amine molecules were protecting the surface of the crystals^19^, the phosphine oxide molecules were selectively passivate edge state and enhanced the PL. We also employed confocal time-resolved PL decay measurements to verify the enhanced lifetime of edge states when we used phosphine oxides (Supplementary Fig. 13).

To verify that P=O bound the perovskites via direct chemical linkages and was not merely incorporated nonspecifically alongside the precursor, we conducted a study that combined Raman spectroscopy, solid-state nuclear magnetic resonance (NMR) spectroscopy, Fourier-transform Infrared spectroscopy (FTIR), X-ray photon spectroscopy (XPS) and X-ray diffraction (XRD) measurements. The Raman spectrum of TPPO agreed with the established literature frequency values, and changed significantly upon addition to the PbBr_2_ precursor or to the perovskites (Supplementary Figs. 14 and 15). Additionally, we utilized solid-state ^31^P NMR spectroscopy to investigate the interaction of TPPO with perovskites. We observed chemical shifts of the TPPO-precursor (TPPO-PbBr_2_) and TPPO-perovskite relative to TPPO itself, indicative of changes in the coordination of phosphorus (Fig. 4f)^32^. The narrow NMR peak for the TPPO-perovskite sample indicated that TPPO assumed a single configuration in the sample, as opposed to the broad range of structures evident in the TPPO-precursor (TPPO-PbBr_2_) spectrum. We measured FTIR spectra of perovskite with and without TPPO edge stabilization and compared to those of TPPO itself (Supplementary Fig. 16). The stretching vibration of P=O in TPPO itself appeared at 1182 cm^−1^ and was shifted to 1179 cm^−1^ upon the formation of TPPO-PbBr_2_ in the perovskite edge-stabilized by TPPO. We attribute this to a weakened P=O bond caused by the interaction with Pb^2+^ in the perovskites. In addition to the above observations, the edge-stabilized perovskite also showed additional IR absorption at 723 cm^−1^, indicating the interaction of the phosphine oxide and perovskites (P=O:Pb). We also found that TPPO was incorporated into perovskite films during the spin-coating process when delivered using an anti-solvent. We observed two additional XRD peaks in these films, at 2*θ* *=* 10.11° and 20.22°, corresponding to the diffraction from (TPPO)_2_PbBr_2_ complexes (Supplementary Fig. 17 for the XRD of TPPO-precursor reference)^33^. XPS was used to determine the presence of phosphor and oxygen atoms in TPPO-treated perovskites. XPS core-level photoemission spectra of C 1*s*, Pb 4*f*, P 2*p*, O 1*s* and Br 3*d* are shown in Supplementary Fig. 18. The results reveal the existence of P in the TPPO-precursor and edge-stabilized perovskites.

### Device performance and operational stability

**Fig. 5:**
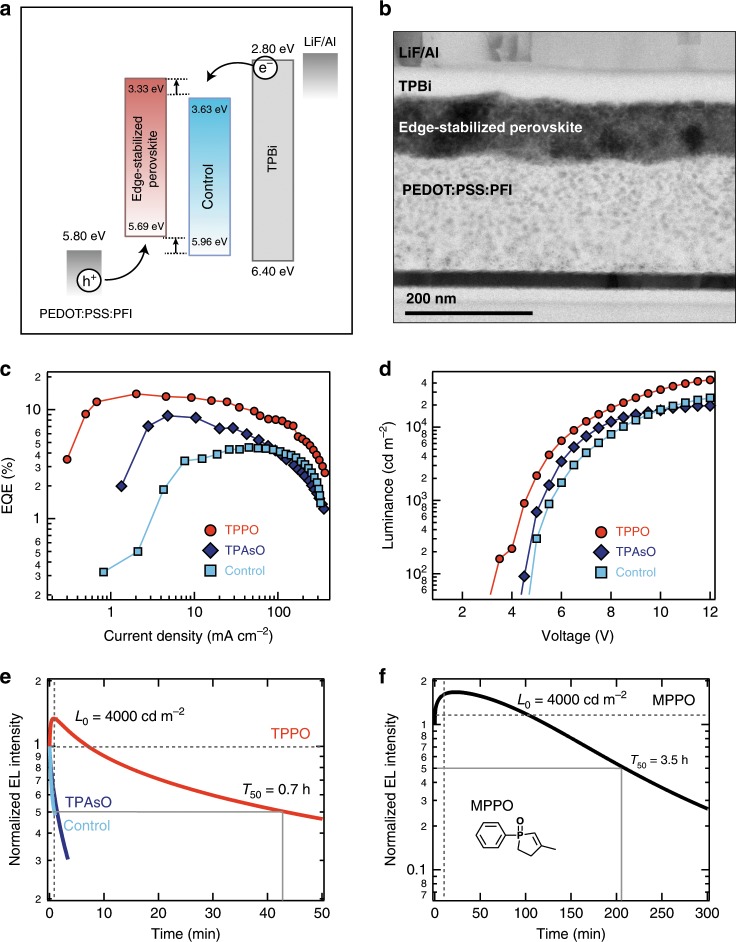
LED performance and operational stability. **a** Cross-section focused ion beam (FIB) transmission electron microscope (TEM) image. **b** Energy band diagram based on literature and UPS measurements. **c** EQE versus current density and **d** luminance versus voltage characteristics of untreated controls and edge-stabilized perovskite LEDs. **e** Operational device stability of untreated controls and edge-stabilized perovskite LEDs at a starting luminance of 4000 cd m^−2^. **f** Device operational stability of the perovskite LED with MPPO at a starting luminance of 4000 cd m^−2^.

We then sought to translate the bright and stable perovskite films into high-efficiency LEDs. We used a device architecture consisting of ITO/PEDOT:PSS:PFI/Perovskite/TPBi/LiF/Al (Fig. 5a, b) (ITO: Indium Tin Oxide; PEDOT:PSS:PFI: poly(3,4-23122−1−122+−122 ethylenedioxythiophene)polystyrene sulfonate doped with perfluorinated ionomer; LiF: Lithium Fluoride). We used PEDOT:PSS:PFI as the hole transport layer in view of its exciton-buffering and hole-injection capabilities^34^, together with 1,3,5-tris(*N*-phenylbenzimiazole-2-yl)benzene (TPBi) as the electron transport layer, and LiF/Al as the cathode. Ultraviolet photoemission spectroscopy (UPS) measurements were used to determine the valence band positions and work functions of the control and edge-stabilized perovskites (Fig. 5a and Supplementary Fig. 19). The work function of the samples was obtained from the ultraviolet radiation energy (21.2 eV), and the energies at secondary cut-offs of UPS spectra. Valence band ionization energy (IE) decreased from 6.55 to 6.14 eV in the edge-stabilized perovskites, due to the electronic structure change caused by surface modification. This enables a reduction in the injection barrier of electrons and holes within the devices. We then measured electron and hole only devices to evaluate the charge injection balance in devices. The results showed that in devices, the edge-stabilized perovskites exhibited a higher balance in electron and hole transport than the untreated control (Supplementary Fig. 20). Also, the electroluminescence (EL) spectra did not change in either cases (Supplementary Fig. 21).

We selected TPPO and TPAsO to fabricate the perovskite layer. We found that compared with the untreated control, the perovskites treated with TPPO or TPAsO showed significantly improved EL performance (Fig. 5c, d and Table 1). The perovskite treated with TPPO exhibited a maximum EQE of 14% and luminance of 4.5 × 10^4^ cd m^−2^ (corresponding to an EQE 5%) (Supplementary Figs. 22–24), exceeding the one treated with TPAsO with an EQE of 9% and maximum luminance of 2.0 × 10^4^ cd m^−2^ (corresponding to an EQE of 2%). In addition, the current density–voltage–luminance (*J–V–L*) measurements at various scan rates and directions attest to an absence of hysteresis (Supplementary Figs. 25, 26). Perovskites were protected by PEA ligands and also by the TPPO as edge ligands, and these may help to slow ion migration.

**Table 1:** Device performance and operational stability of LEDs based on perovskites with and without edge stabilization.

Perovskites	PLQY (%)	*V*_on_ (V)	Max EQE (%)	Max *L* (cd m^−2^)	*T*_50_ at 4000 cd m^−2^	*T*_50_ at 100 cd m^−2^
No edge stabilization	40	3.5	4.5	26,700	53 s	11 min
TPAsO	92	4.5	8.8	19,990	82 s	12 min
TPPO	98	3.5	14.0	45,230	44 min	33 h

Since the physically absorbed oxygen inside the perovskites contributes to severe photoelectric degradation even in encapsulated devices, operational instability remains a critical issue in perovskite LEDs^9^. We therefore explored whether the edge-stabilization strategy could enhance device operational stability. The LEDs were biased to achieve an initial luminance of 4000 cd m^−2^; we then studied the variation in their EL intensity (Fig. 5e). Control perovskites with no edge stabilization, as well as the perovskites treated with TPAsO, lost 50% of the initial emission within 53 and 82 s, respectively, while the perovskite treated with TPPO showed a longer half-lifetime (*T*_50_) of 44 min. Similar stability trends were also observed at a lower initial luminance of 100 cd m^−2^ (Table 1). We therefore designed another molecule, 3-methyl-1-phenyl-2-phospholene 1-oxide (MPPO), which had a smaller steric hindrance, and could bind Pb more effectively. We achieved a much longer *T*_50_ of 3.5 h at 4000 cd m^−2^ (Fig. 5f and Supplementary Fig. 27). Comparing among all the previous reports, including those that reported lifetimes only at low luminance, this work provides the highest brightness ever observed in a long-lived LED (initial luminance of 3800 cd m^−2^, with *T*_50_ = 10 min)^20^. Moreover, we attribute a sharp degradation of the device after 3.5 h operation to the interface-induced chemical reaction between the perovskite and charge transport layer that accelerates the materials degradation rather than to perovskite degradation itself. The interfacial contact between perovskite/TPBi and LiF/Al has been reported to be another critical factor limiting operational stability. The edge-stabilized perovskites retained the PL intensity in air over 300 min on ITO/ZnO/PVP, but exhibited faster decay (lose 25% initial PL in 5 min) on PEDOT:PSS:PFI layers (Supplementary Fig. 28). The acidic nature of PEDOT:PSS caused corrosion of the active materials, highlighting the importance of further device interface engineering to improve stability.

## Discussion

In summary, we demonstrate an edge-stabilization strategy that achieves bright and stable reduced-dimensional perovskites with high PLQYs and suppressed photodegradation. We incorporate phosphine oxides during film fabrication and then passivate otherwise exposed layer edges. The resulting perovskites exhibit a remarkable robustness against oxygen, moisture and heat. When implemented as active layers in LEDs, they showed a peak EQE of 14%, maximum luminance of 4.5 × 10^4^ cd m^−2^, and an operational half-lifetime of 3.5 h at 4000 cd m^−2^ under continuous operation. This is 21 times longer than the best green LEDs previously reported. Our edge-stabilization strategy can be applied to other types of perovskites, including quantum dots and polycrystalline films with a range of emission wavelengths.

## Methods

### Fabrication of perovskite films

In PEA_2_Cs_2.4_MA_0.6_Pb_4_Br_13_ perovskite, precursors PbBr_2_ (0.6 M) (99.999% Alfa-Aesar), CsBr (0.36 M) (99.999%, Sigma-Aldrich), MABr (0.1 M) (Dyesol) and PEABr (0.3 M) (Dyesol) were dissolved in DMSO. The precursor was spin-coated onto a glass substrate using a two-step method^35^. During the second step of the spin-coating process, 100–500 µL of chloroform was dropped onto the substrate. For the edge-stabilized perovskite films, TPPO (98%, Sigma-Aldrich) was dissolved in chloroform (5–10 mg mL^−1^) and deposited onto the perovskite film during the second step. The resulting films were then annealed at 90 °C for 7 min to increase crystallization.

### Device fabrication and characterization

A mixed solution of PEDOT:PSS (Clevios^TM^ PVP Al4083) and perfluorinated ionomer, tetrafluoroethylene-perfluoro-3,6-dioxa-4-methyl-7-octenesulfonic acid copolymer (PFI) (PEDOT:PSS:PFI = 1:6:25.4 (mass ratio)), was spin-coated on the ITO-coated glass substrates treated with oxygen-plasma; and this was followed by an annealing step on a hot plate at 150 °C for 20 min in air^36^. Perovskite precursor solutions were spin-coated onto the PEDOT:PSS-coated substrate via the two-step anti-solvent spin-coating method. TPBi (60 nm) and LiF/Al electrodes (1 nm/100 nm) were deposited using a thermal evaporation system under a high vacuum of <10^−4^ Pa. The device active area was 6.14 mm^2^ as determined by the overlap between the ITO and Al electrodes. LEDs were encapsulated prior to measurement. All devices were tested in ambient conditions (Supplementary Fig. 30).

We developed an experimental setup for organic LED measurements based on a procedure adapted from Forrest et al.^37^. The angular dependence of intensity and EL spectrum are taken into consideration via the use of the integrating sphere, which collects emitted light across angles. The current density–voltage (*J–V*) characteristic was measured using a Keithley 6430 source meter. The absolute radiation flux for calculating the EQE, power efficiency and luminance was collected with a measurement system containing an integrating sphere and an Ocean Optics USB4000 spectrometer, which was calibrated with a standard halogen lamp (Ocean Optics HL-2000). The devices were mounted on the open aperture of the integrating sphere to allow the light emitted from the glass surface to be collected; while the emission from the substrate edges was not collected. When calculating the luminance, a Lambertian emission profile was assumed (Supplementary Fig. 31). The integration time for the spectrometer was set for 10 ms in order not to saturate the CCD detector during measurement. We have reproduced the measurements multiple times and also for several repetitions of the same experiment. The device was encapsulated for the efficiency measurement with a UV-resin (exposure under UV light for 10 s) and covered on the device and a transparent glass substrate.

We used half-lifetime (*T*_50_), the time after which the device luminance drops to 50% of the initial value, to describe the device operational stability. The half-lifetime measurements were carried out in a nitrogen-filled glovebox without encapsulation. Devices were driven by a Keithley 2400 source meter at constant current, and the luminance intensity was measured using a commercial photodiode (Vishay Semiconductors BPW34). The current density used to drive the LEDs was first determined using the EQE measurement system. To achieve a high initial luminance of 4000 cd m^−2^, the current densities were: control devices: 25 mA cm^−2^; TPAsO-treated: 15 mA cm^−2^; and TPPO-treated 8 mA cm^−2^. To achieve an initial luminance of 100 cd m^−2^, the current density for the LEDs were: control 2.5 mA cm^−2^ and TPAsO 1.5 mA cm^−2^. For the LEDs based on MPPO, we used 10 mA cm^−2^ to achieve an initial luminance of 4000 cd m^−2^. The photodiode was biased at 0 V and the photocurrent, proportional to the luminance, was recorded at 2 s intervals.

### Photoluminescence measurement

A Horiba Fluorolog system was used for PL measurements. Steady-state PL was measured with a monochromatized Xe lamp as the excitation source. A Time Correlated Single Photon Counting detector and a pulsed UV laser diode (*λ* = 374 nm) were used to acquire transient PL. An instrument response function of Δ*t* ~0.13 ns limits the overall time resolution. Time-resolved emission spectra were recorded by measuring individual transient PL traces at increasing emission wavelengths. PLQYs were obtained by coupling a Quanta-Phi integrating sphere to the Fluorolog system through optical fiber bundles. All PLQY measurements followed published methods^38^. We measured the both excitation and emission spectra, where the sample was directly excited by the excitation beam path in the integrating sphere, the sample offset within the integrating sphere from the beam path; and we also measured the empty sphere itself in order to acquire information regarding the excitation beam. We include raw spectra of near-unity PLQY data from edge-stabilized perovskite in Supplementary Fig. 32. The PL spectrum of perovskite controls and edge-stabilized perovskites reveal emission centered at 517 nm, with the edge-stabilized perovskite films showing full-width at half-maximum of 22 nm compared to 28 nm for the control. To investigate the role of TPPO on the passivation of edge traps, we carried out temperature-dependent PL measurements. As the temperature decreases, the PL intensity of unpassivated perovskites steadily increases, indicative of trap freezing that results in reduced trap recombination losses. The PL intensity of the edge-stabilized perovskites, on the other hand, remains unchanged, suggesting negligible trapping even at room temperature. This result agrees with the measured near-unity PLQY values of edge-stabilized perovskites (97 ± 2%) compared to the case of control perovskite samples (60 ± 10%) and the radiative decay time of edge-stabilized perovskites (Supplementary Fig. 33). The PL stability test was performed with a nanosecond pulsed diode laser, with continuous 374 nm excitation at 1 mW cm^−2^ power density. All perovskite films were tested in air with relatively low humidity. The photothermal stability test was performed by placing the sample on an aluminum block with a resistive heater. A thermocouple and temperature controller were used to maintain active feedback. The sample was excited using a 442 nm diode laser. The light was then coupled to a spectrometer (USB200+).

### Transmission electron microscopy

High-resolution HAADF-STEM images were acquired using a probe-corrected FEI Titan microscope operating at 300 kV. A probe semiconvergence angle of ~20 mrad was used. Due to the sensitivity of layered perovskites to the electron beam, a reduced dose below 10 e A^−2^ s^−1^ was used during the imaging.

### Device cross-section focused ion beam TEM

Transmission electron microscopy (TEM) samples were prepared using an FEI Helios NanoLab 400S focused ion beam (FIB)/SEM dual-beam system equipped with a Ga+ ion source. C/Pt layers were deposited on the surface region of interested via electron and ion beam for protection. The sample was thinned to 80 nm lamella using progressively decreasing ion beam energies in the FIB down to 2 keV.

### GIWAXS measurements

GIWAXS measurements were performed at beamline 7.3.3 at the Advanced Light Source, Lawrence Berkeley National Laboratory. The wavelength of the X-ray beam employed was 1.24 Å. The scattering patterns were obtained at a photon incidence angle of 0.25° with respect to the sample plane. Samples were scanned in a He environment to reduce air scattering. Exposure times were typically between 5 s and 30 s. The scattering patterns were recorded using a Pilatus 2M detector at a fixed distance of 277.674 mm. Calibration of the lengths in reciprocal space was carried out by using silver behenate. A heating stage was set up for temperature-dependent in situ studies. The GIWAXS scans were taken from 90 °C to 150 °C. The annealing temperature was increased by 10 °C at a time, and kept at 150 °C for 30 min. After the temperature elevating process, the samples were cooled down to 50 °C. Samples for GIWAXS were spin-coated on glass substrates following the same spin coating and annealing procedures as were used in fabricating LEDs.

### ^31^P NMR measurements

The samples were finely ground, then packed evenly into 4 mm zirconia rotor, and sealed at the open end with a Vespel cap. The ^31^P NMR spectra were acquired using a Bruker 400 MHz AVANACIII NMR spectrometer equipped with double resonance Bruker MAS probe (BrukerBioSpin, Rheinstetten, Germany). The spectra were recorded using onepulse pulse program with 14 kHz spinning rate. To achieve high signal to noise ratio, the spectra were recorded by collecting at least 1 k scans with recycle delay time of 10 s. Bruker Topspin 3.2 software (Bruker BioSpin, Rheinstetten, Germany) was used to record the NMR spectra and to analyze the data.

^31^P NMR was used to study of the tri-octylphosphine chalconide moieties capping the surface of CdSe nanocrystals. We observed solid-state ^31^P NMR chemical shifts in TPPO-perovskite compared to bare TPPO, indicative of changes in the coordination of phosphorus^39^. The narrow NMR peak for the edge-stabilized perovskite sample indicates that TPPO assumes a single configuration in the sample, in contrast to the broad range of structures evident in the TPPO-precursor spectrum. The weakening of the P=O bond signal upon the coordinating to the metal surface presented in ^31^P NMR measurement. The increase of chemical shift in TPPO-perovskite has been observed from a decrease in the electron density at phosphorous due to the oxygen coordination with Pb. The signal in NMR spectroscopy can also depend on the crystal facet to which the element is adsorbed. A peak broadening in TPPO-precursor sample would suggest the presence of species on different surfaces.

### DFT simulations

Calculations were performed using the Quickstep module of the CP2K computational package^40^, using a MOLOPT double-zeta plus polarized orbital basis set^31^, Goedecker–Teter–Hutter pseudopotentials^32^, grid cut-off of 600 Ry, and Perdew–Burke–Ernzerhof exchange-correlation functional^33^. Layered lead bromide perovskites with *n* = 3 were modeled using Cs as a cation both inside and on the surface for computational efficiency. To represent better the bandgaps and level alignments of the molecules with perovskite, free molecules were computed using the B3LYP functional, using a Cl_2_ molecule as a common energy reference level. The results for the molecule + perovskite calculated at the PBE level are consistent with the findings from B3LYP for free molecules^34^.

A supercell made of 4 × 3 × 3 orthorhombic (Pnma) unit cells was constructed, with only Γ k-point used for simulations. The slabs are separated by 30 Å of vacuum in the *z*-direction, made periodic in the *x*-direction, and expose unpassivated edges along the *y*-direction, with 20 Å between the periodic images. All geometries were relaxed until forces on atoms converged to below 40 meV Å^−1^, including the cell-size degrees of freedom.

The edges in the *y*-direction are cut along the (110) direction of the orthorhombic cell (corresponding to (100) direction in cubic notation), in agreement with TEM images of the CsPbBr_3_ nanoplatelets and colloidal nanocrystals^41,42^. The edges are saturated by Cs Br (or PEA Br) and do not expose any Pb. However, slabs prepared in such a way have an overall excess of cations, which leads to either charging or the electronic doping of such systems which in turn become prone to the desorption of excess cations (Cs or PEA). The first candidate for desorption is Cs at the corners, but the charge balance requires even more cations to be desorbed, leading to openings along the edges. One can also expect that a desorption of a charge-neutral CsBr or PEABr can be possible, especially near the already exposed site with one ligand lost, and as a result exposing one more Pb. All three types of defects expose one dangling bond of Pb, which is susceptible to molecular adsorption. DMSO, TPPO and O_2_ molecules were adsorbed onto the remaining exposed Pb dangling bond. Binding energies were calculated as a difference between *E*_surf+molecule_, *E*_surf_ and *E*_molecule_in_gas_phase_. Entropy effects were not computed as they typically do not exceed 0.1 eV.

## Supplementary information

Supplementary Information

## Data Availability

The data that support the findings of this study are available from the corresponding author upon reasonable request.
