# Supplementary material for: Edge stabilization in reduced-dimensional perovskites
Source: Nat Commun. 2020 Jan 10;11:170. doi: 10.1038/s41467-019-13944-2 (PMC6954198; doi:10.1038/s41467-019-13944-2)
Supplement: Supplementary file 1 — Supplementary Information [file 41467_2019_13944_MOESM1_ESM.pdf]

Supplementary Information for

Edge stabilization in reduced-dimensional perovskites

Li Na Quan,[1†] Dongxin Ma,[1†] Yong-Biao Zhao,[2,1†] Oleksandr Voznyy,[1] Haifeng Yuan,[3,1] Eva Bladt,[4] Jun Pan,[7] F. Pelayo García de Arquer,[1] Randy Sabatini,[1] Zachary Piontkowski,[6] Abdul-Hamid Emwas,[8] Petar Todorovic,[1] Rafael Quintero-Bermudez,[1] Grant Walters,[1] James Z. Fan,[1] Mengxia Liu,[1] Hairen Tan,[1] Makhsud I. Saidamino,[1] Liang Gao,[1,10] Yiying Li,[2] Dalaver H. Anjum,[8] Nini Wei,[8] Jiang Tang,[10] David W. McCamant,[6] Maarten B. J. Roeffaers,[5] Sara Bals,[4] Johan Hofkens,[3,9] Osman M. Bakr,[7] Zheng-Hong Lu,[2]* Edward H. Sargent[1]*

[1]Department of Electrical and Computer Engineering, University of Toronto, 10 King's College Road, Toronto, Ontario, M5S 3G4, Canada.

[2]Department of Materials Science and Engineering, University of Toronto, 184 College Street, Toronto, Ontario M5S 3E4, Canada.

[3]Department of Chemistry, KU Leuven, Celestijnenlaan 200F, B-3001 Leuven, Belgium.

[4]EMAT, University of Antwerp, Groenenborgerlaan 171, 2020 Antwerp, Belgium.

[5]Centre for Surface Chemistry and Catalysis, KU Leuven, Celestijnenlaan 200F, B-3001 Leuven, Belgium.

[6]Department of Chemistry, University of Rochester, 120 Trustee Rd., Rochester, NY14627, USA.

[7]Division of Physical Science and Engineering, King Abdullah University of Science and Technology (KAUST), Thuwal 23955-6900, Kingdom of Saudi Arabia

[8]Imaging and Characterization Core Lab, King Abdullah University of Science and Technology, Thuwal 23955-6900, Saudi Arabia.

[9]RIES, Hokkaido University, N20W10, Kita-Ward Sapporo 001-0020 Japan.

[10]Wuhan National Laboratory for Optoelectronics (WNLO), Huazhong University of Science and Technology (HUST), Wuhan 430074, China

† These authors contributed equally to this work.

E-mail: ted.sargent@utoronto.ca; zhenghong.lu@utoronto.ca

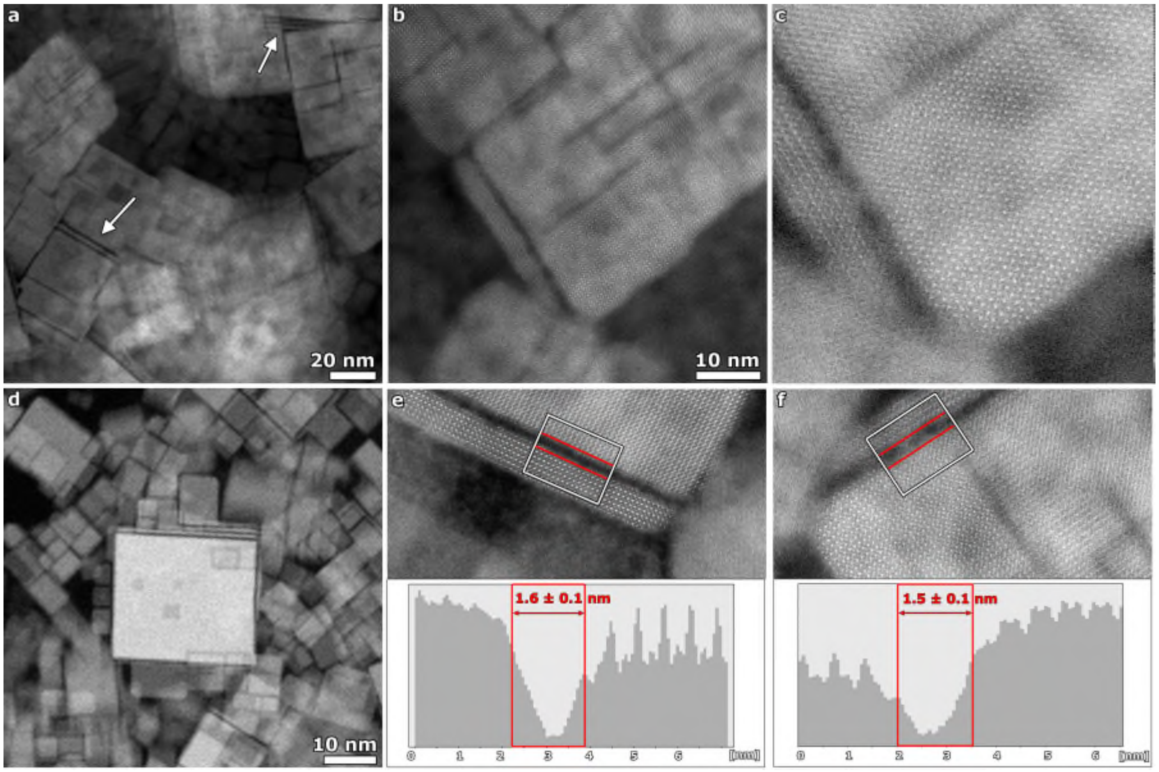

Supplementary Figure 1 | HAADF-STEM images of layered perovskites film. (**a**) HAADF-STEM image where the presence of layered perovskites is indicated by the white arrows. (**b,c**) High resolution HAADF-STEM images of a single sheet of perovskite. (**d**) HAADF-STEM image of a layered perovskite with TPPO showing 3 layers. (**e,f**) High resolution HAADF-STEM images of a layered perovskite, in which the distance between the individual layers is approximately 1.5-1.6 nm. No structural difference observed between perovskite and edge-stabilized perovskite samples.

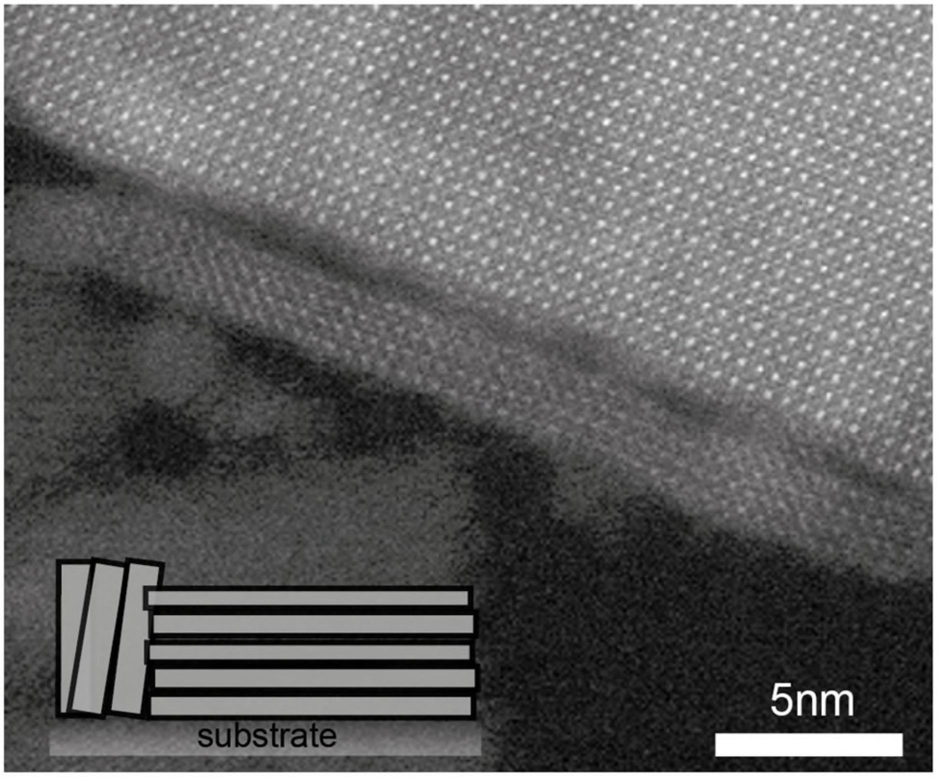

Supplementary Figure 2 | High resolution HAADF-STEM image of a layered perovskite. HAADF-STEM image where the presence of step edges is observed. Due to the electron beam sensitivity of the perovskites, it is uncertain whether these step edges are native characteristics of the perovskite or that these step edges are formed upon electron beam scanning.

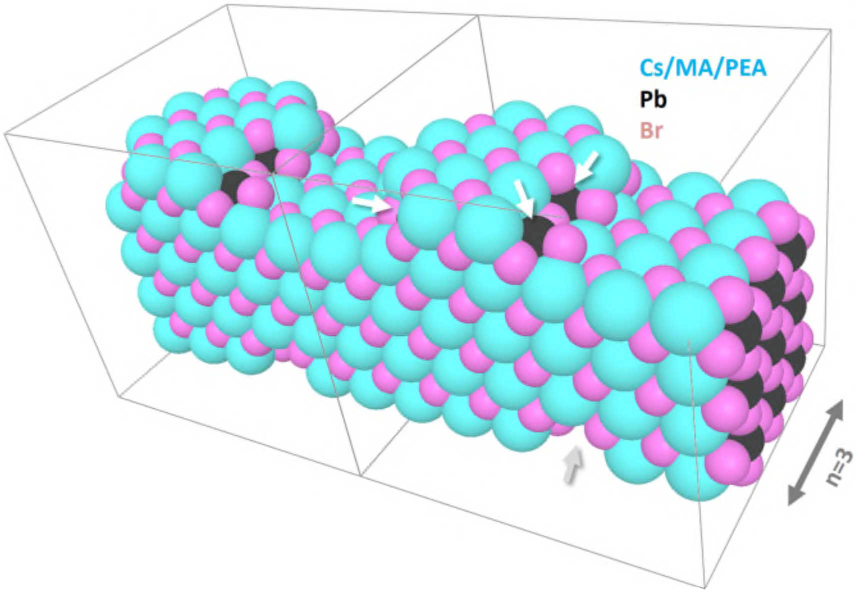

Supplementary Figure 3 | One of the perovskite structures used for DFT calculations (two periodic unit cells are shown).

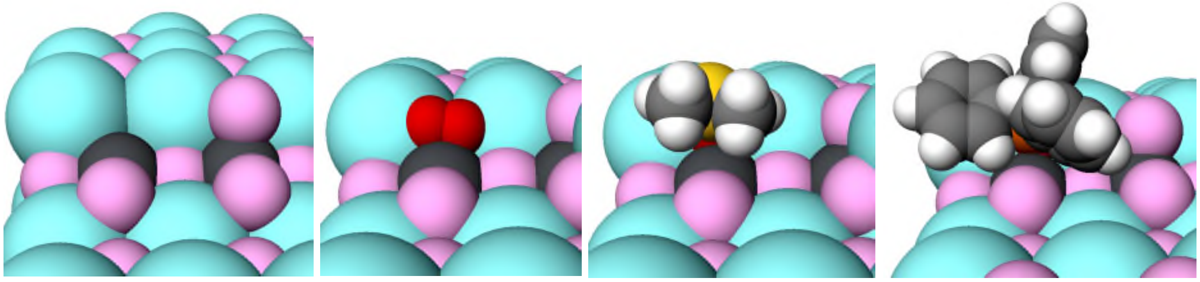

Supplementary Figure 4 | Relaxed geometry of adsorbed molecules (2, oxygen; 3, DMSO; 4, TPPO) on perovskite structure.

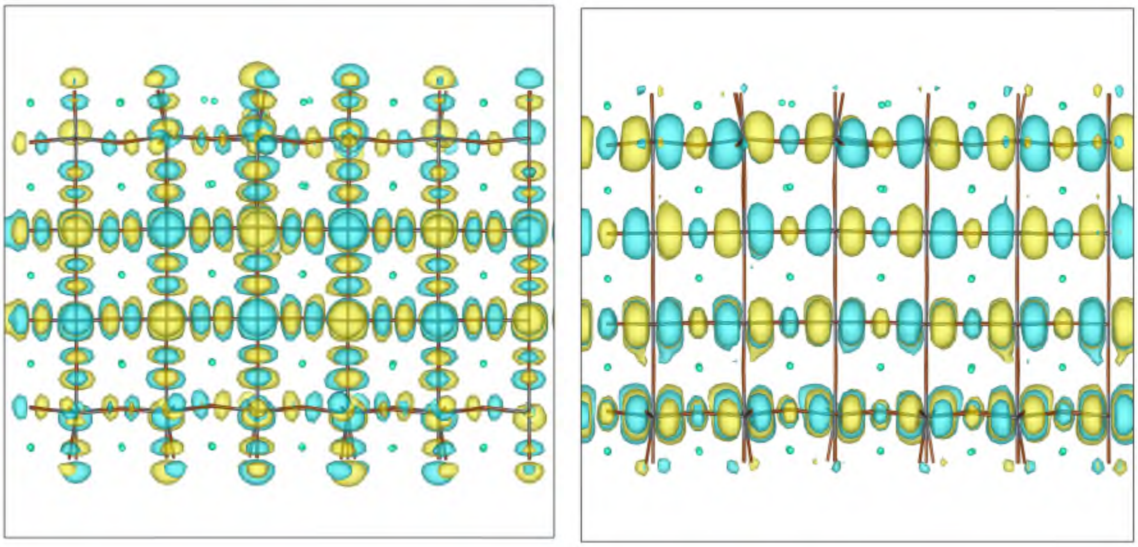

Supplementary Figure 5 | HOMO and LUMO states delocalized on the whole slab, confirming that the Pb dangling bonds do not form any trap states (states localized on the edge / dangling bonds).

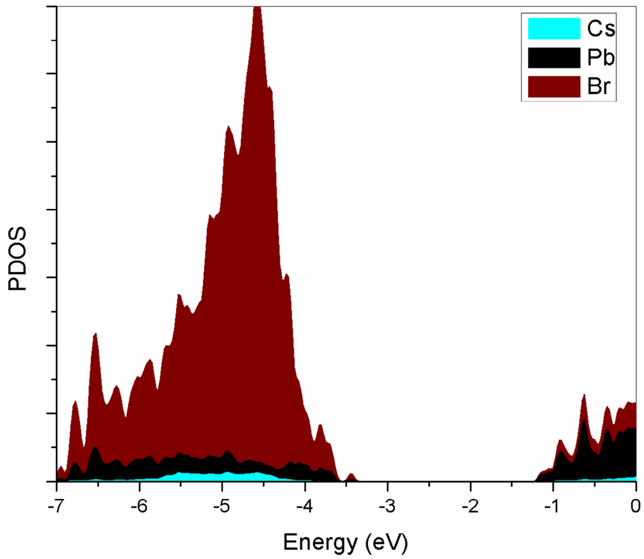

Supplementary Figure 6 | Projected density of states plot showing a clean bandgap for the chosen edge reconstruction.

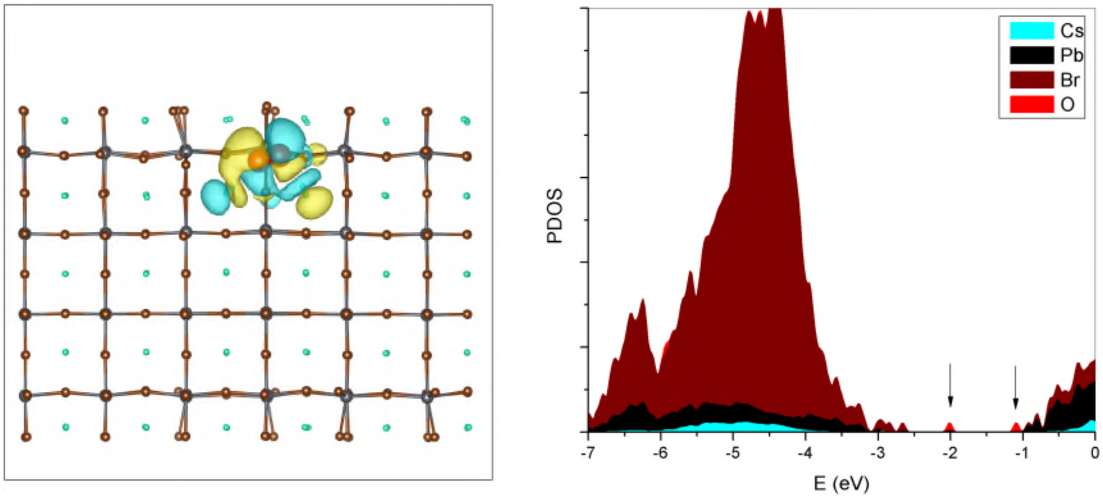

Supplementary Figure 7 | Adsorption of molecular oxygen on the Pb dangling bond exposed due to desorption of Cs/PEA results in localized states within the bandgap (traps) that deteriorate the optoelectronic properties of this material.

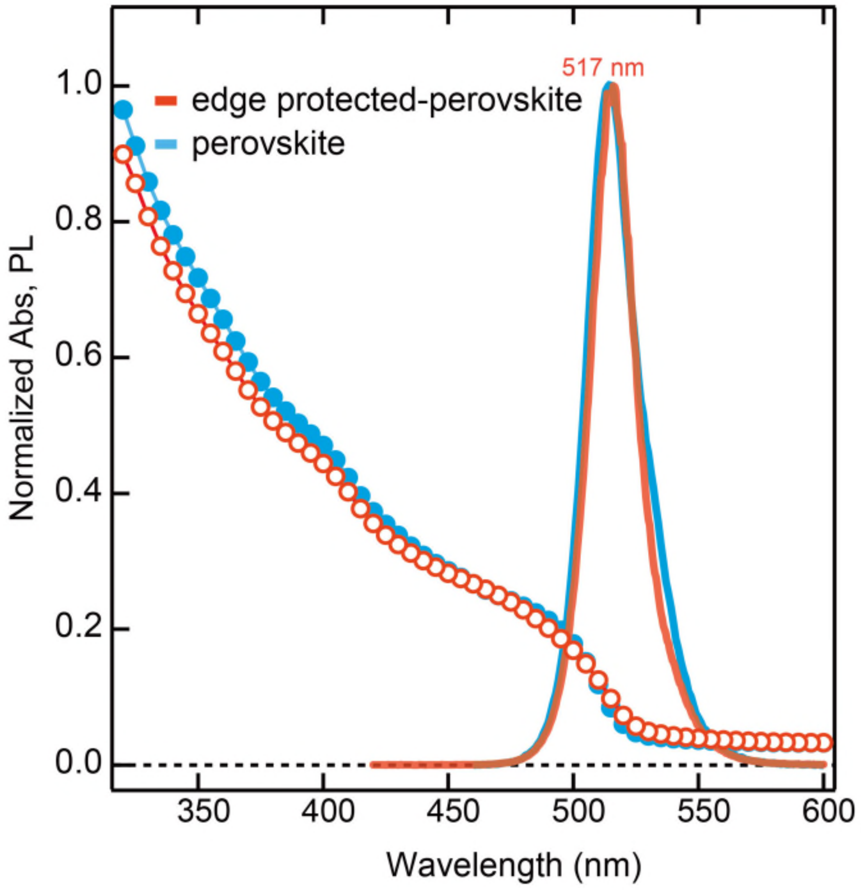

Supplementary Figure 8 | Absorption and PL spectra of perovskite and edge-stabilized perovskite films.

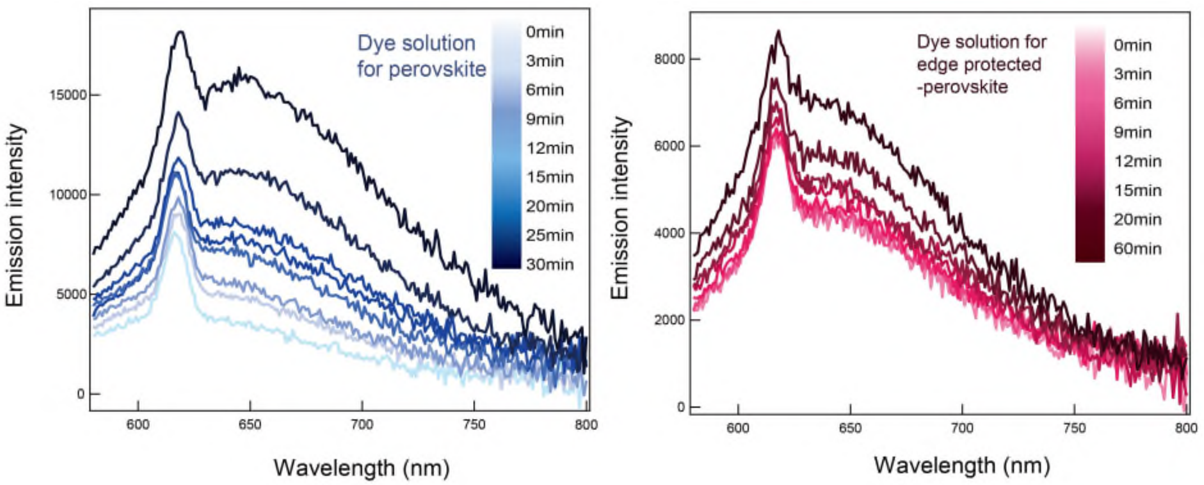

Supplementary Figure 9 | Original emission curves of dye solution, intensity observed from the ageing under the UV illumination and dry air.

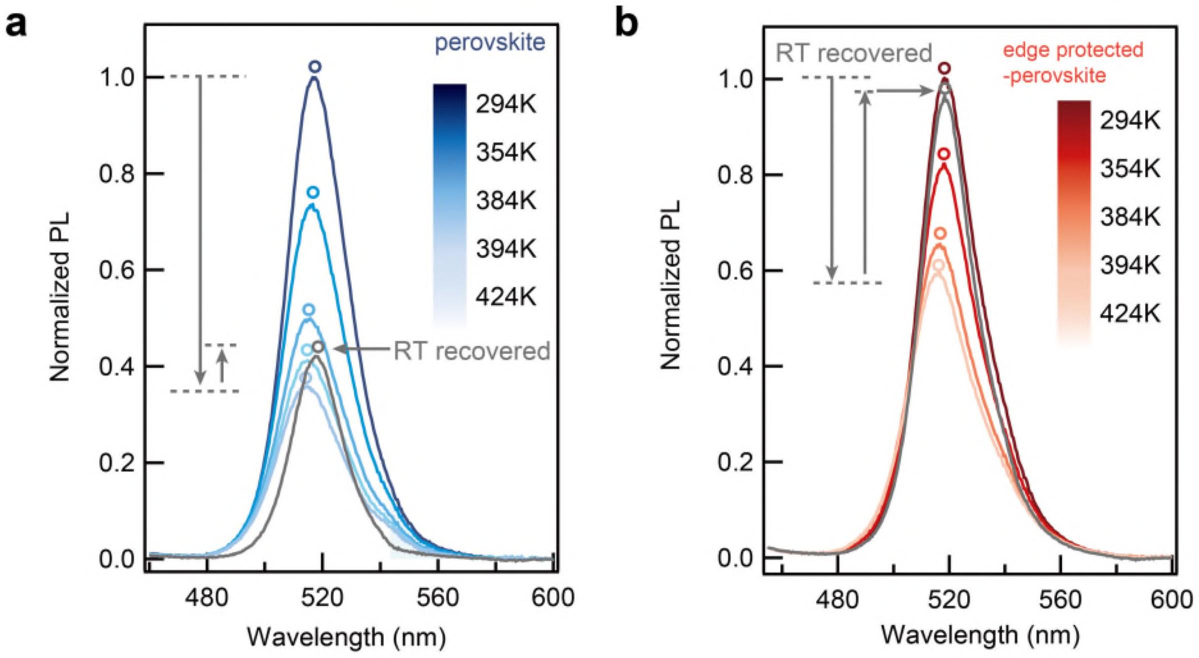

Supplementary Figure 10 | PL spectra of thermal stability test for (a) perovskite and (b) edge-stabilized perovskites.

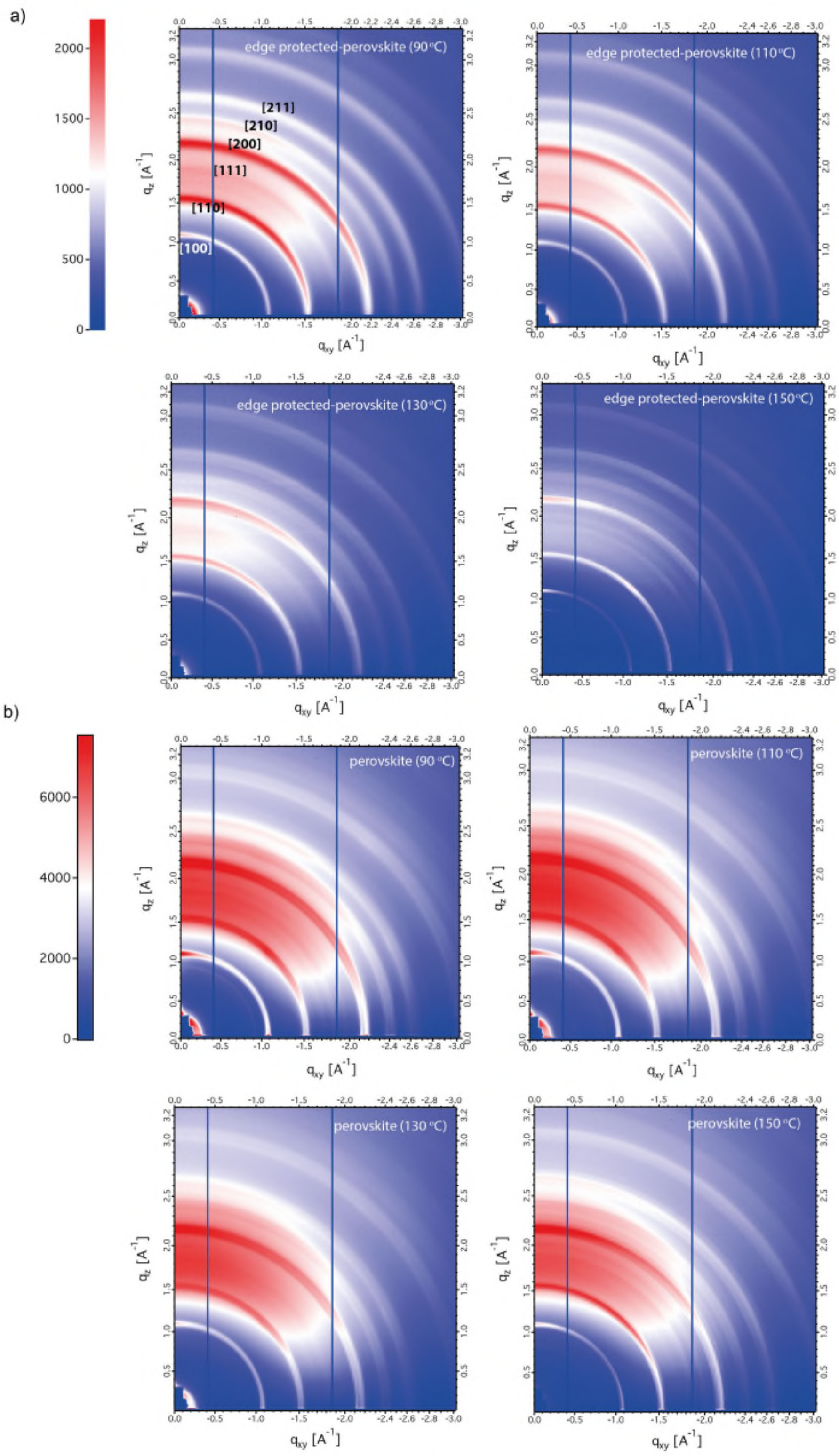

Supplementary Figure 11 | In-situ GIWAX study of (a) edge-stabilized perovskite and (b) perovskite films after continuous heat stress from 90 °C to 150 °C for 30min.

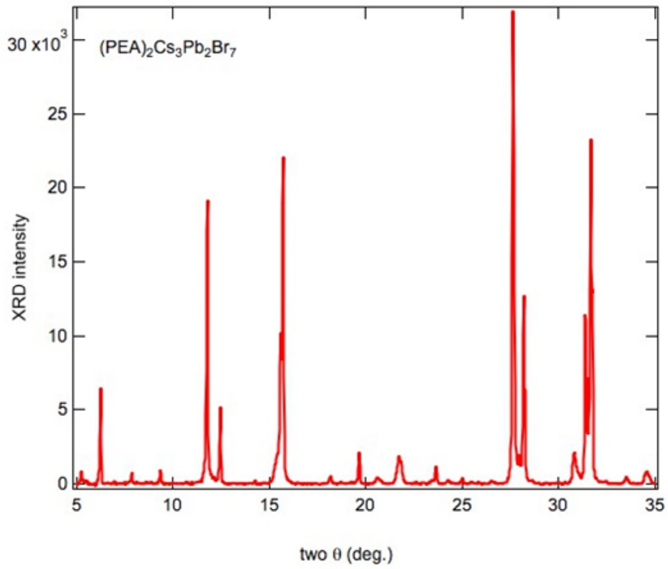

Supplementary Figure 12 | XRD spectra from ground $(PEA)_2Cs_3Pb_2Br_7$ crystal powders.

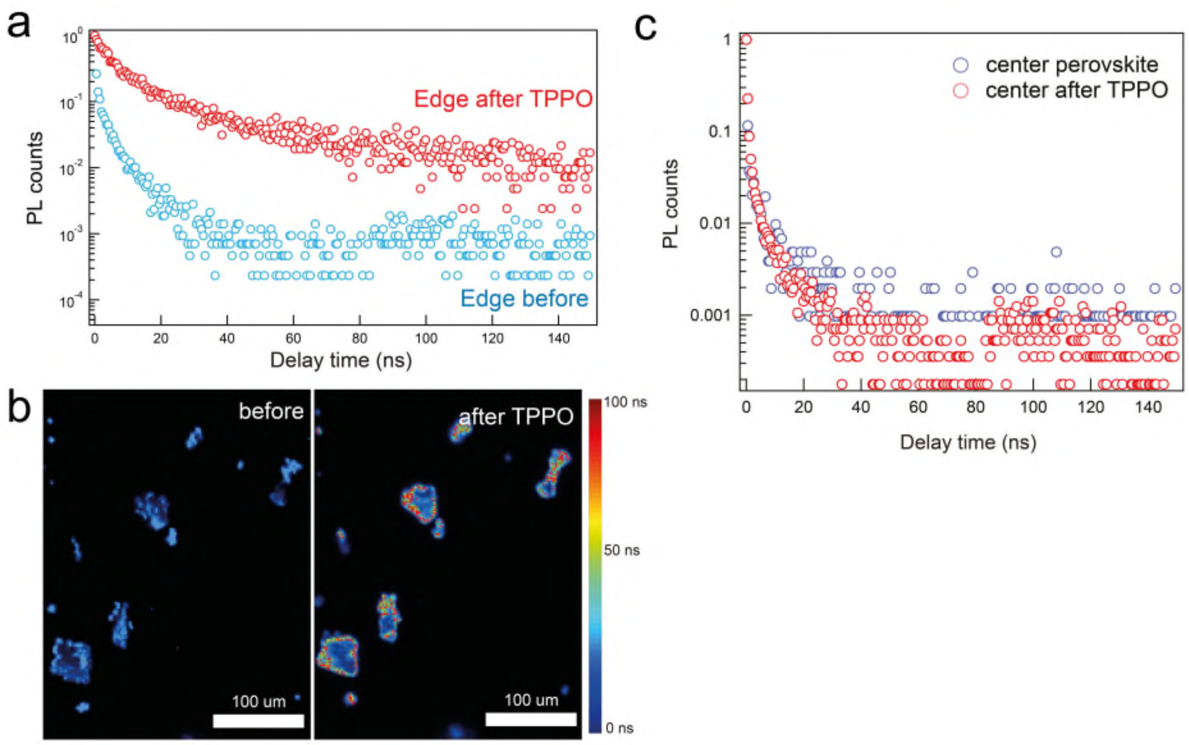

Supplementary Figure 13 | (**a**) Photoluminescence decay plots for the same edge position of a crystal before and after TPPO treatments. (**b**) Photoluminescence decay map of exfoliated perovskite crystals before and after TPPO treatment. Red to blue color in scale bar indicates photoluminescence decay time from 100 ns to 0 ns. (**c**) PL decay spectra mapping for center of exfoliated perovskite crystal before and after TPPO treatment.

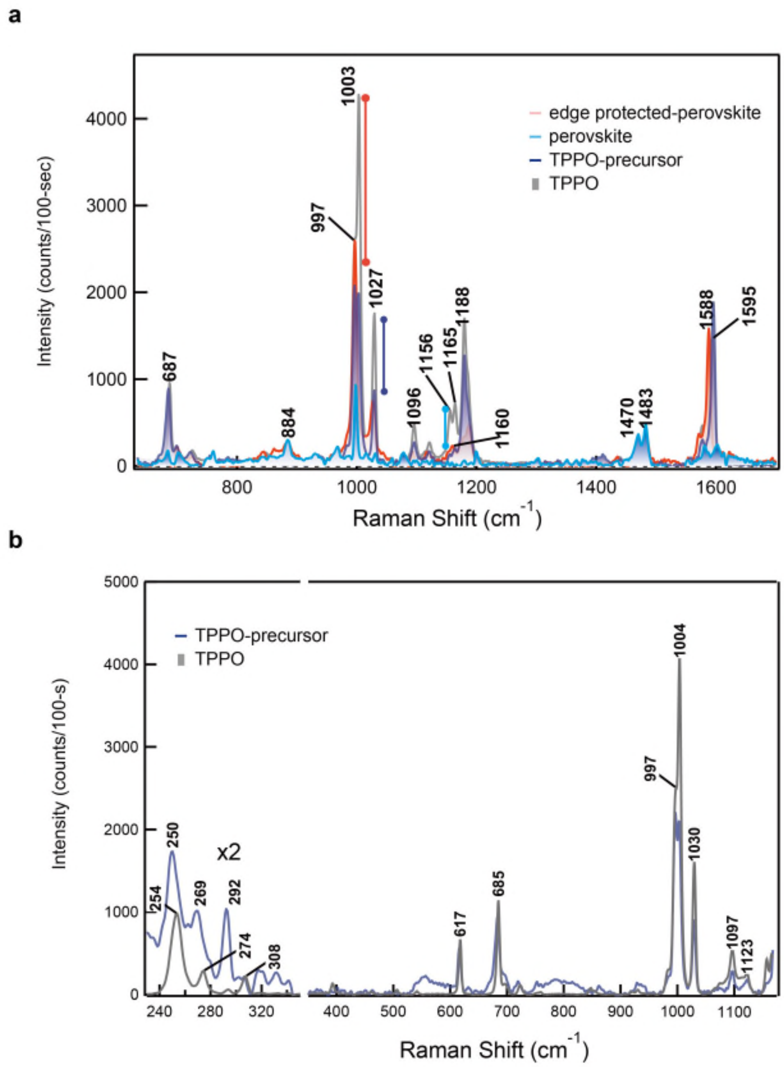

Supplementary Figure 14 | Raman spectra of edge-stabilized perovskite, perovskite, TPPO-precursor films and TPPO powders. Like the TPPO-precursor spectrum, the two peaks become nearly equal in intensity, compared to the TPPO spectrum where the 1003 cm^{-1} mode is larger by a factor of ~1.6. The intensity of the peaks around 1160 cm^{-1} decreases even more than in TPPO-precursor, in which they only decreased by ~25%. There are large frequency shifts in the low-frequency peaks of TPPO at 254, 274 and 308 cm^{-1} compared to the TPPO-precursor sample. Raman spectra were collected with a 561 nm Raman system.

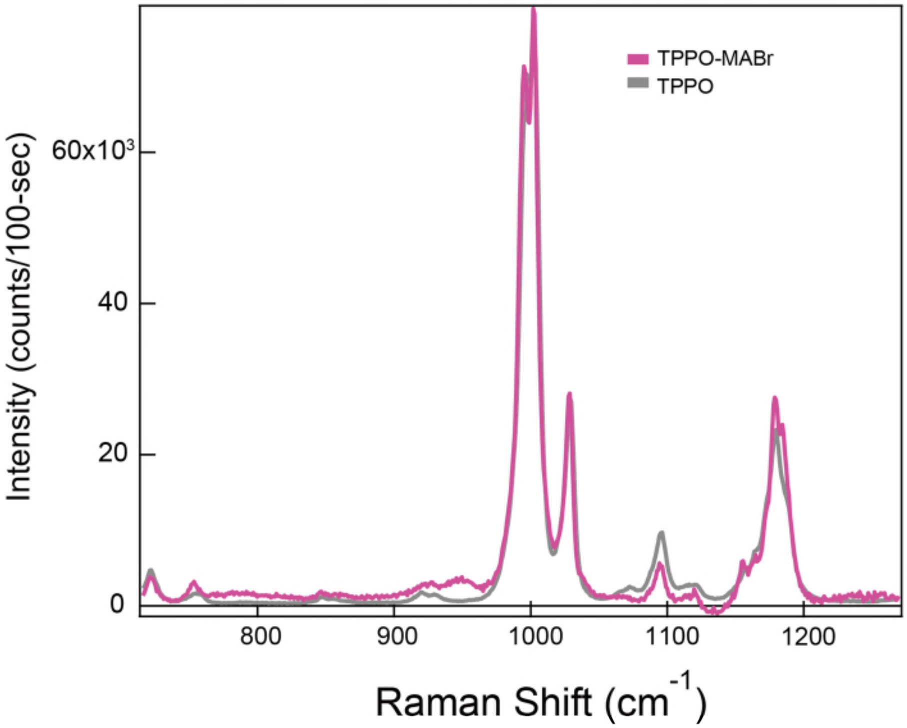

Supplementary Figure 15 | Raman spectra of TPPO and TPPO-MABr powders, to confirm no significant interaction between TPPO and MABr. Spectrum collected with 785-nm Raman system.

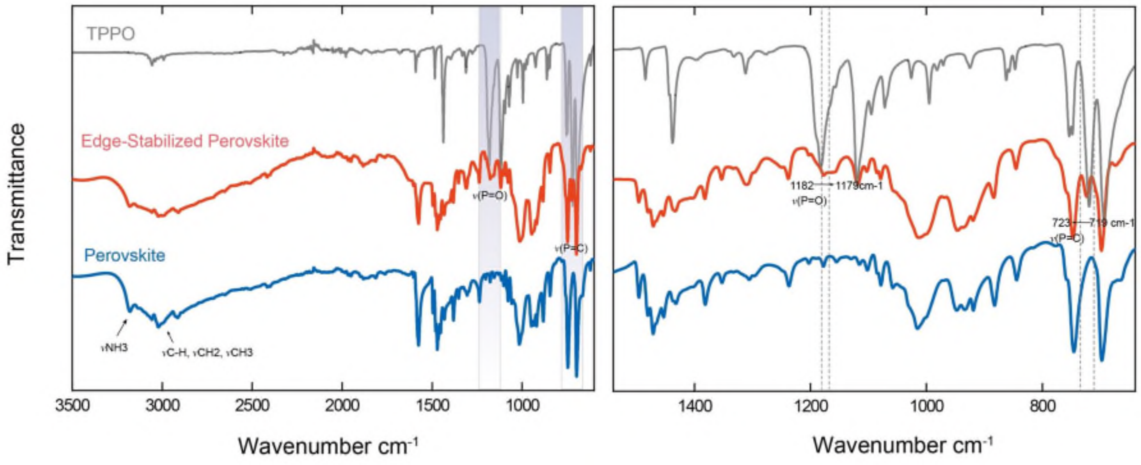

Supplementary Figure 16 | FTIR spectrum of TPPO, edge-stabilized perovskite and control perovskites.

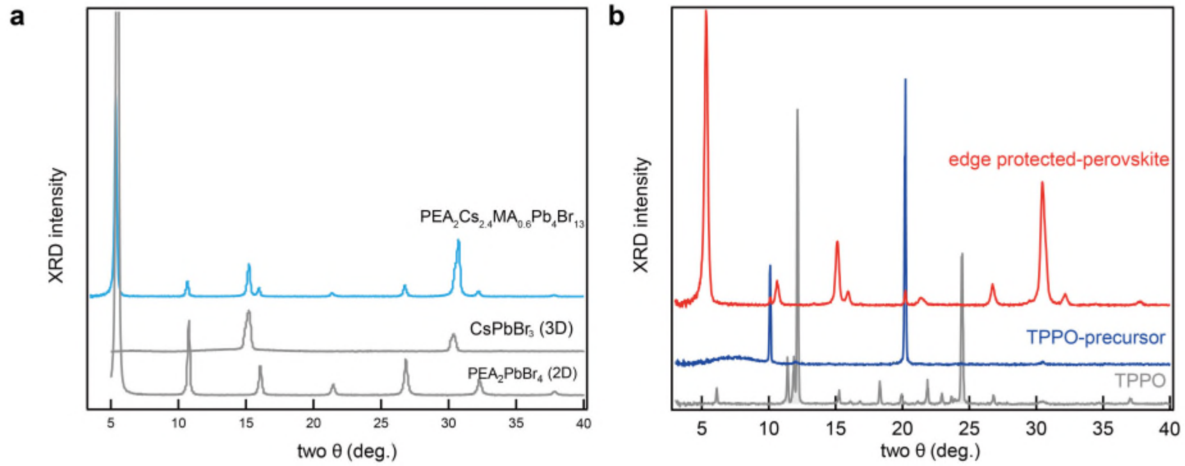

Supplementary Figure 17 | Characterization of perovskite structure. (**a**) XRD spectra of layered perovskite $PEA_2Cs_{2.4}MA_{0.6}Pb_4Br_{13}$, 3D perovskite $CsPbBr_3$ and 2D perovskite PEA_2PbBr_4 films. (**b**) XRD spectra of quasi-2D TPPO-perovskite $PEA_2Cs_{2.4}MA_{0.6}Pb_4Br_{13}$, TPPO-precursor (TPPO-$PbBr_2$) films and TPPO powder.

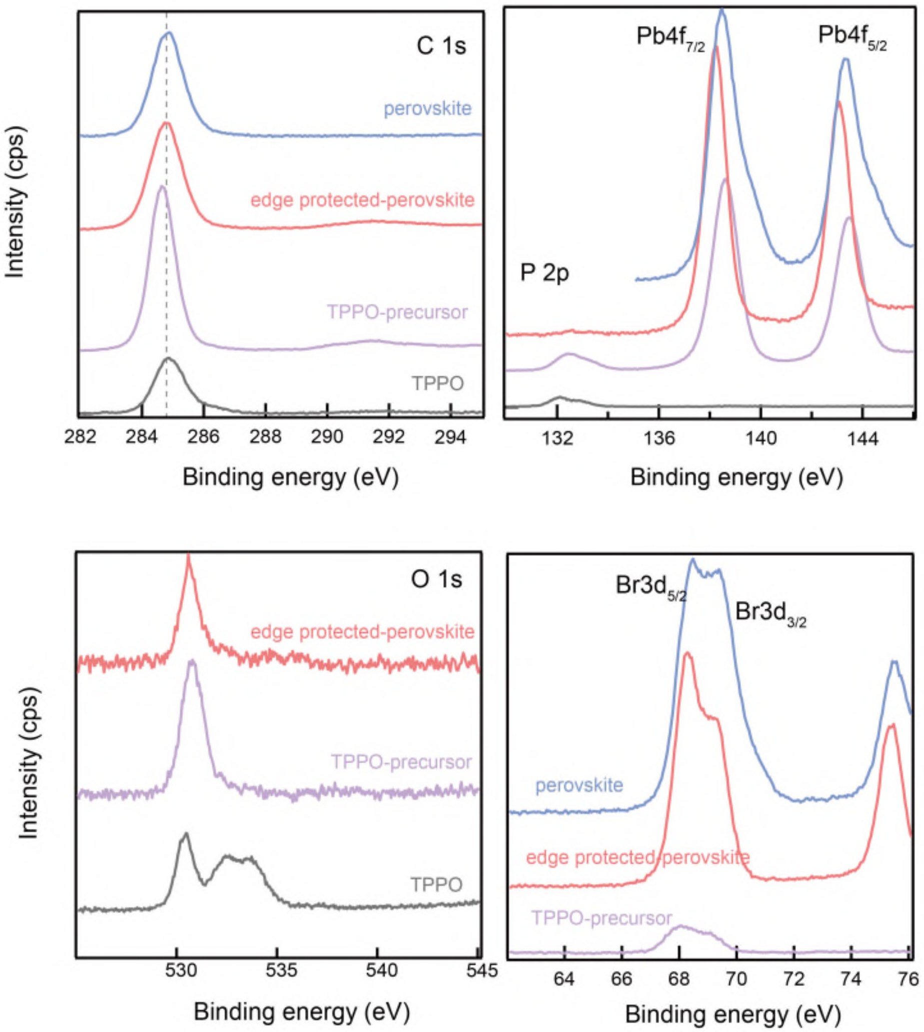

Supplementary Figure 17 | XPS spectra of C1s, P2p, Pb4f, O1s and Br3d in perovskite, edge-stabilized perovskite, TPPO-precursor and TPPO samples.

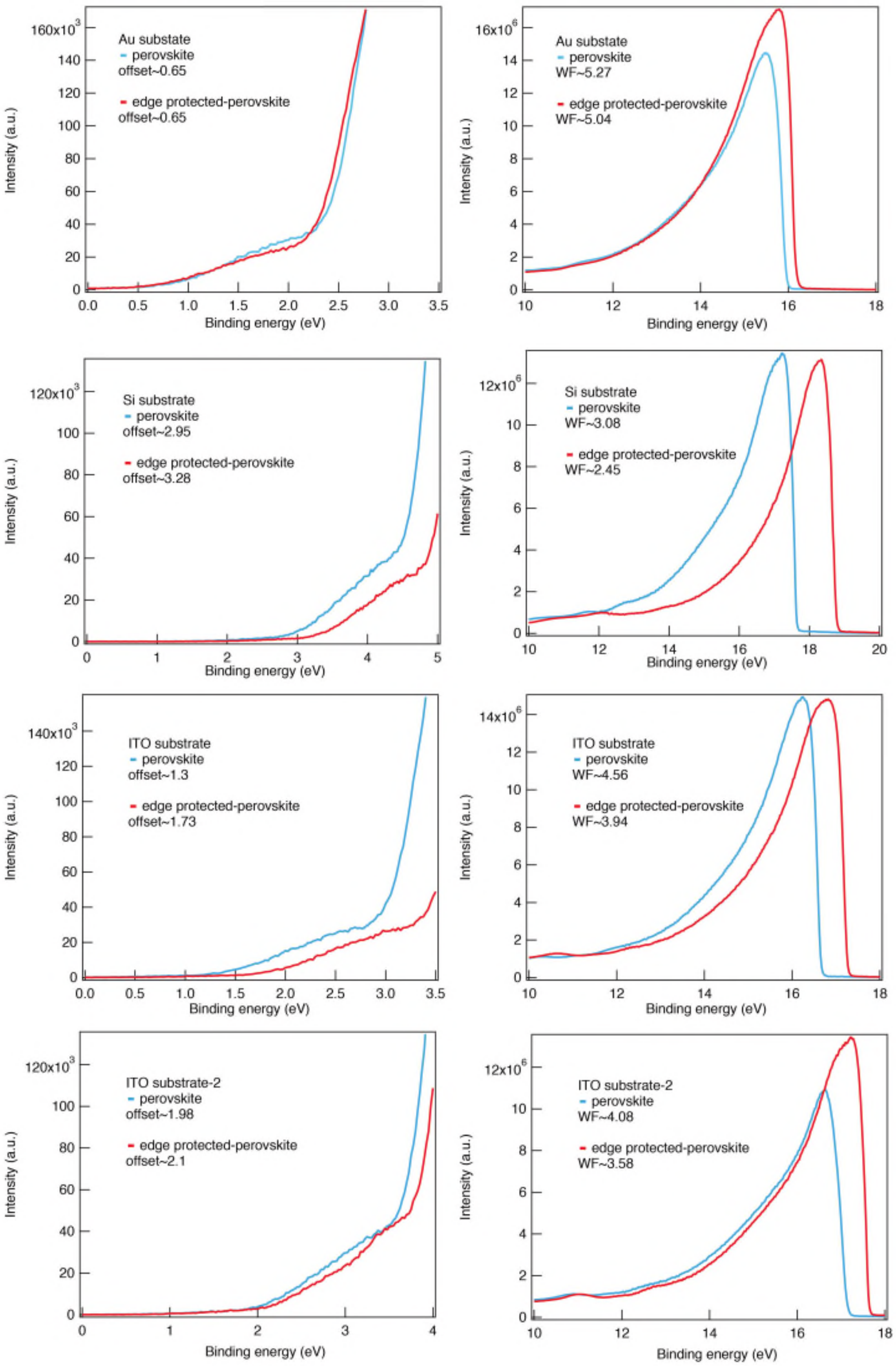

Supplementary Figure 19 | UPS spectra of (a) secondary cut-offs and (b) offset between WFs and IEs in perovskite and edge-stabilized perovskites on the different substrates.

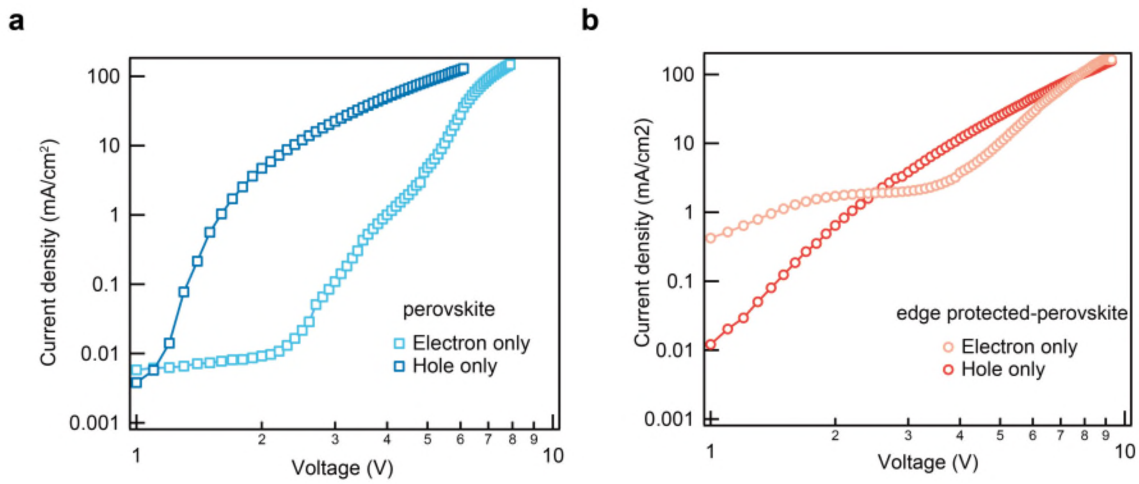

Supplementary Figure 20 | Current density (J_{sc})-Voltage (V) curve of electron and hole only device with perovskite (a) and edge-stabilized perovskites (b).

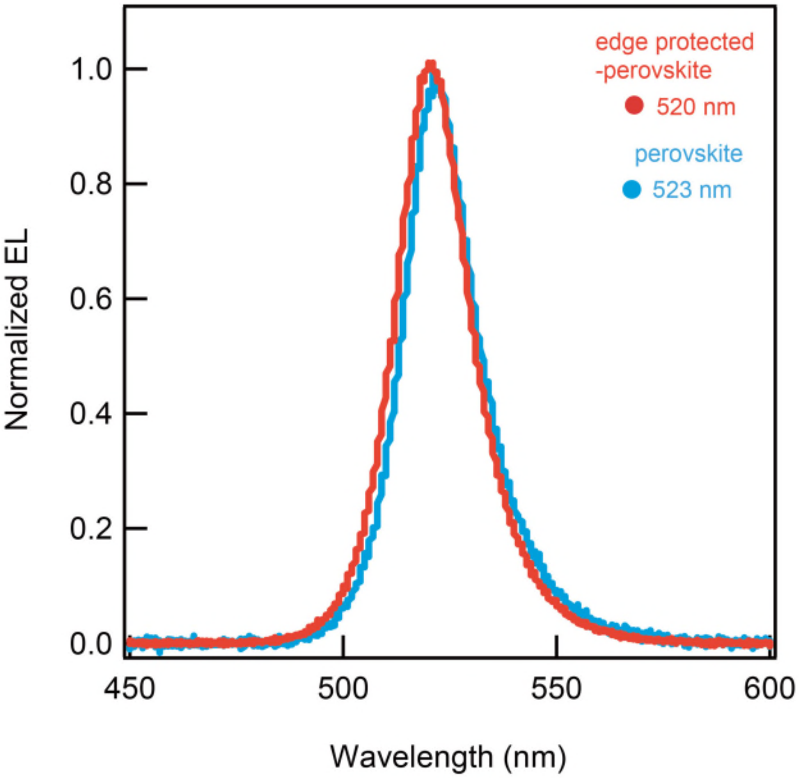

Supplementary Figure 21 | EL spectra of perovskite and edge-stabilized perovskite LEDs.

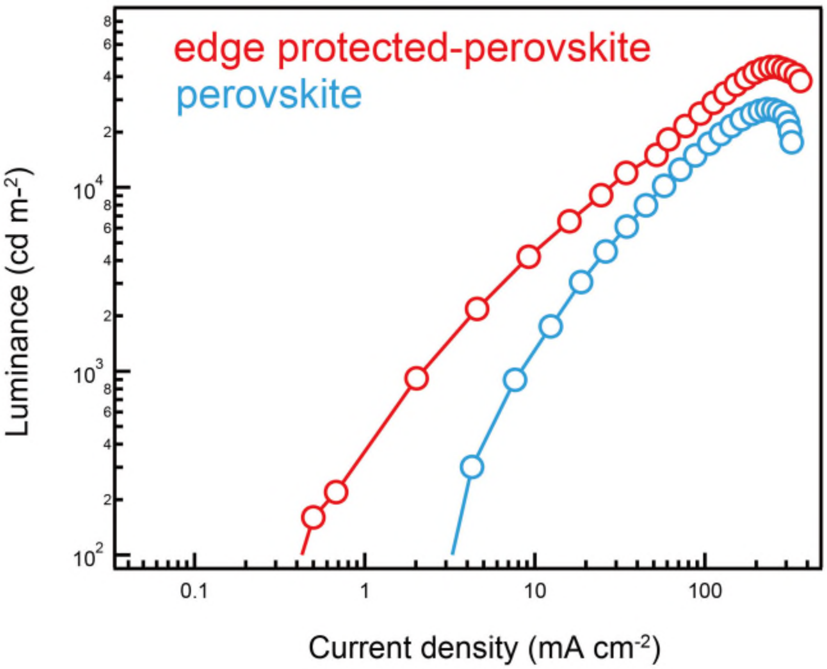

Supplementary Figure 22 | Luminance *vs* current density curve for the with and without edge-stabilized perovskite devices.

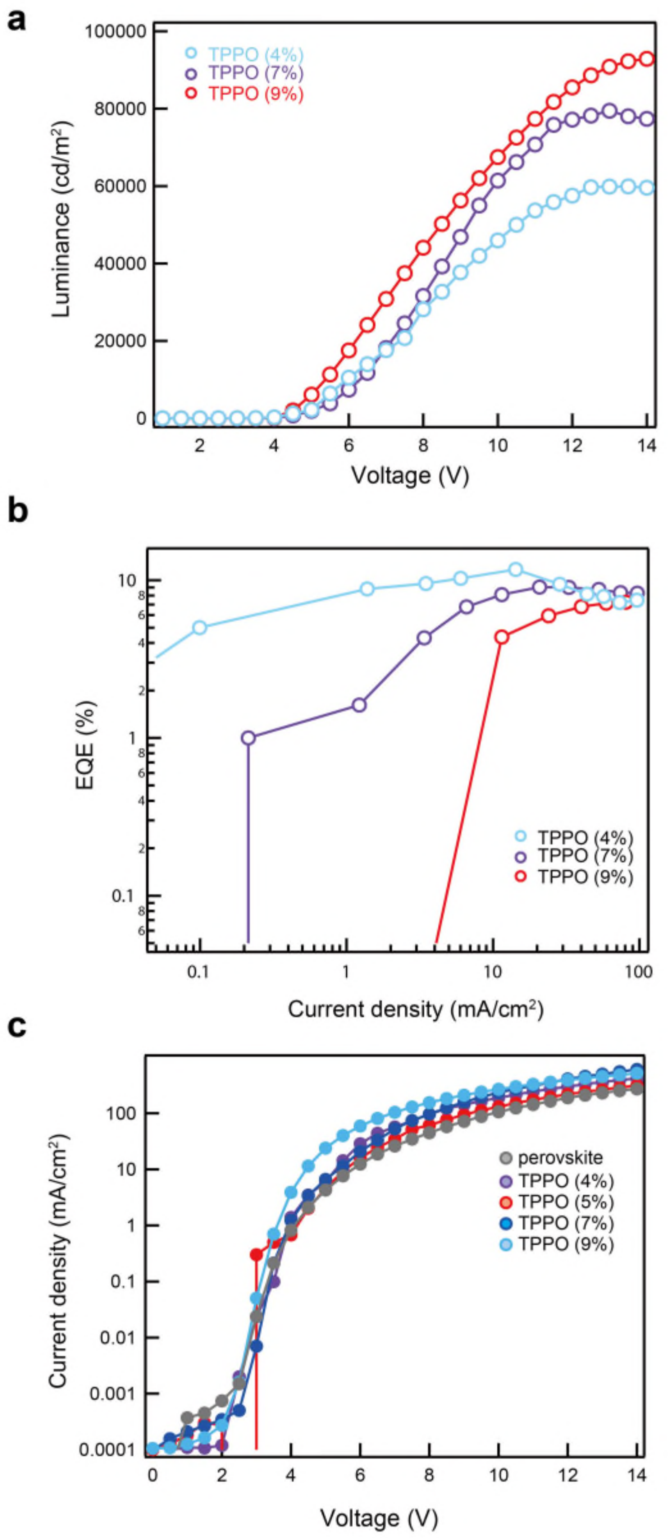

Supplementary Figure 23 | LED performance of edge-stabilized perovskites (a-b) and current density-voltage characteristics (c). We varied the TPPO concentration in the perovskite film in order to maximize the device efficiency.

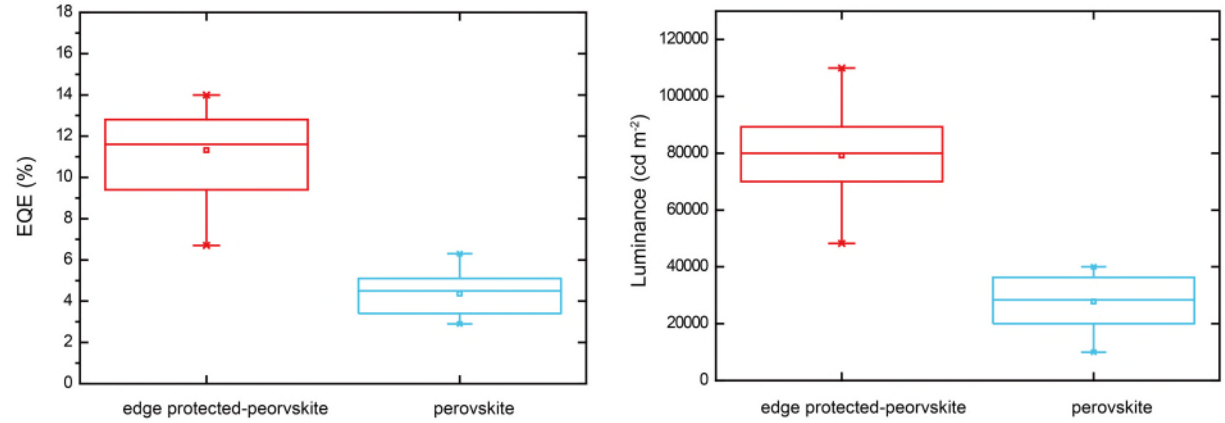

Supplementary Figure 24 | Device performance statics for more than 50 devices.

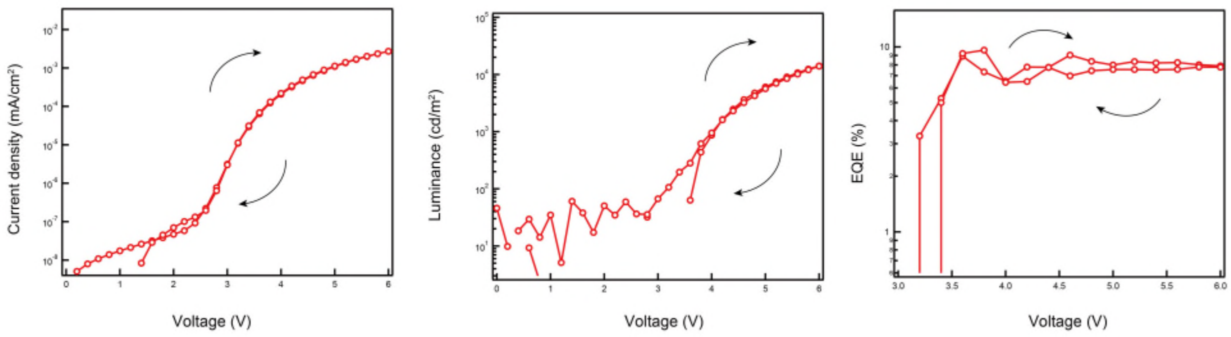

Supplementary Figure 25 | Device hysteresis characterization of edge-stabilized perovskite LEDs. Scanning rate of 0.9 V/s.

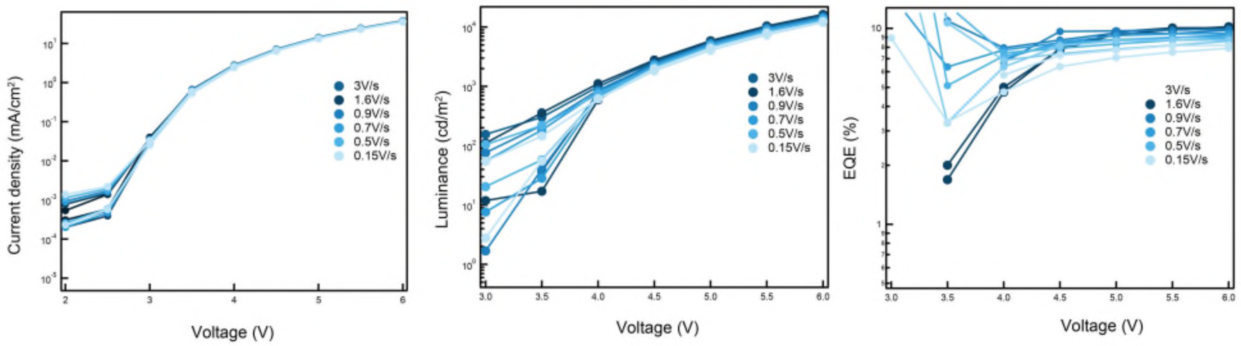

Supplementary Figure 26 | Device hysteresis measurement with different scan rate.

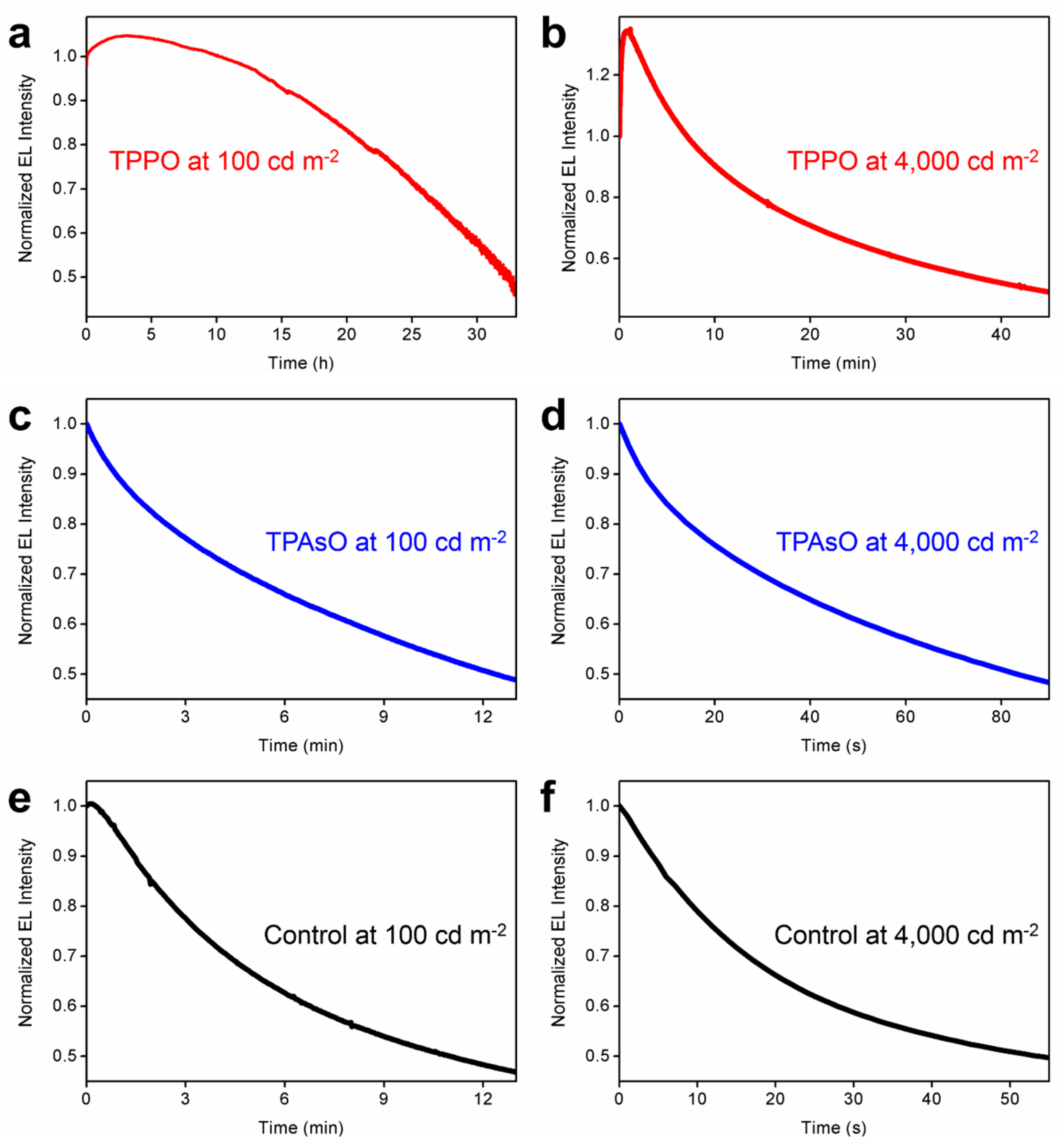

Supplementary Figure 27 | Device operational stability. Stability of LEDs based on the perovskite edge-stabilized with TPPO at (**a**) 100 cd m^{-2} and (**b**) 4,000 cd m^{-2}. Stability of LEDs based on the perovskite edge-stabilized with TPAsO at (**c**) 100 cd m^{-2} and (**d**) 4,000 cd m^{-2}. Stability of LEDs based on the untreated control perovskite at (**e**) 100 cd m^{-2} and (**f**) 4,000 cd m^{-2}.

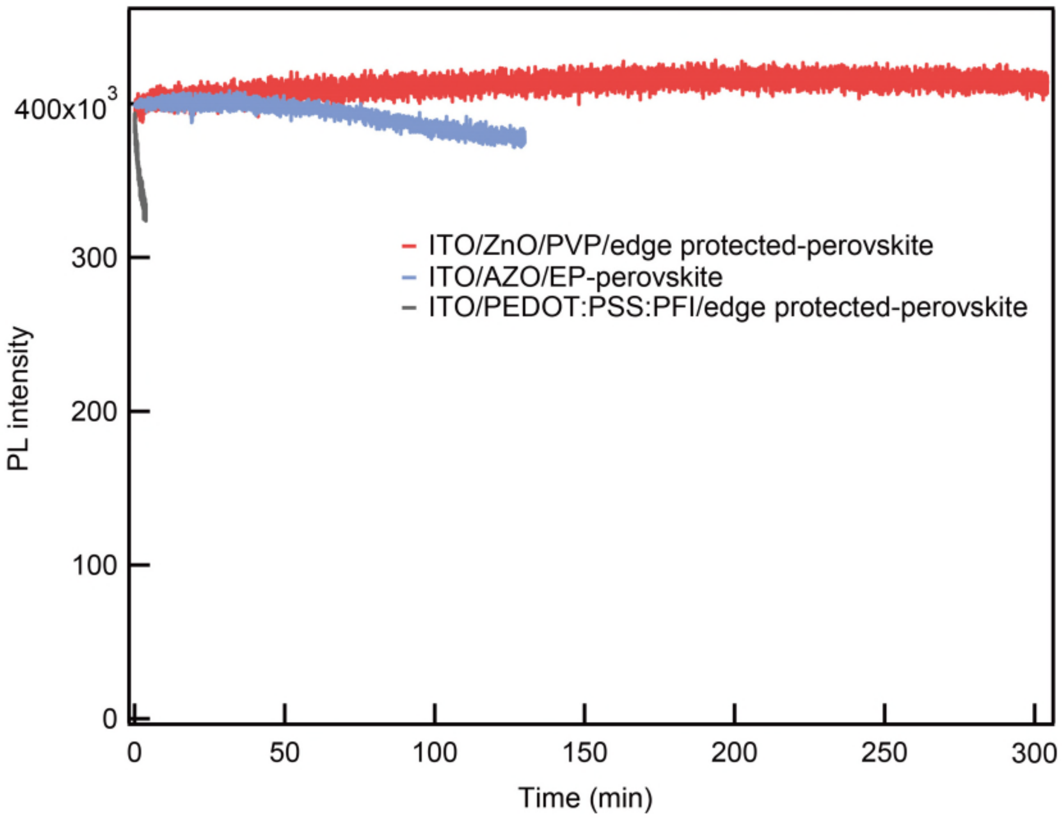

Supplementary Figure 28 | PL stability of edge-stabilized perovskites on the different electrode and charge transport layers under the continuous excitation of 8mW cm^{-2} in air.

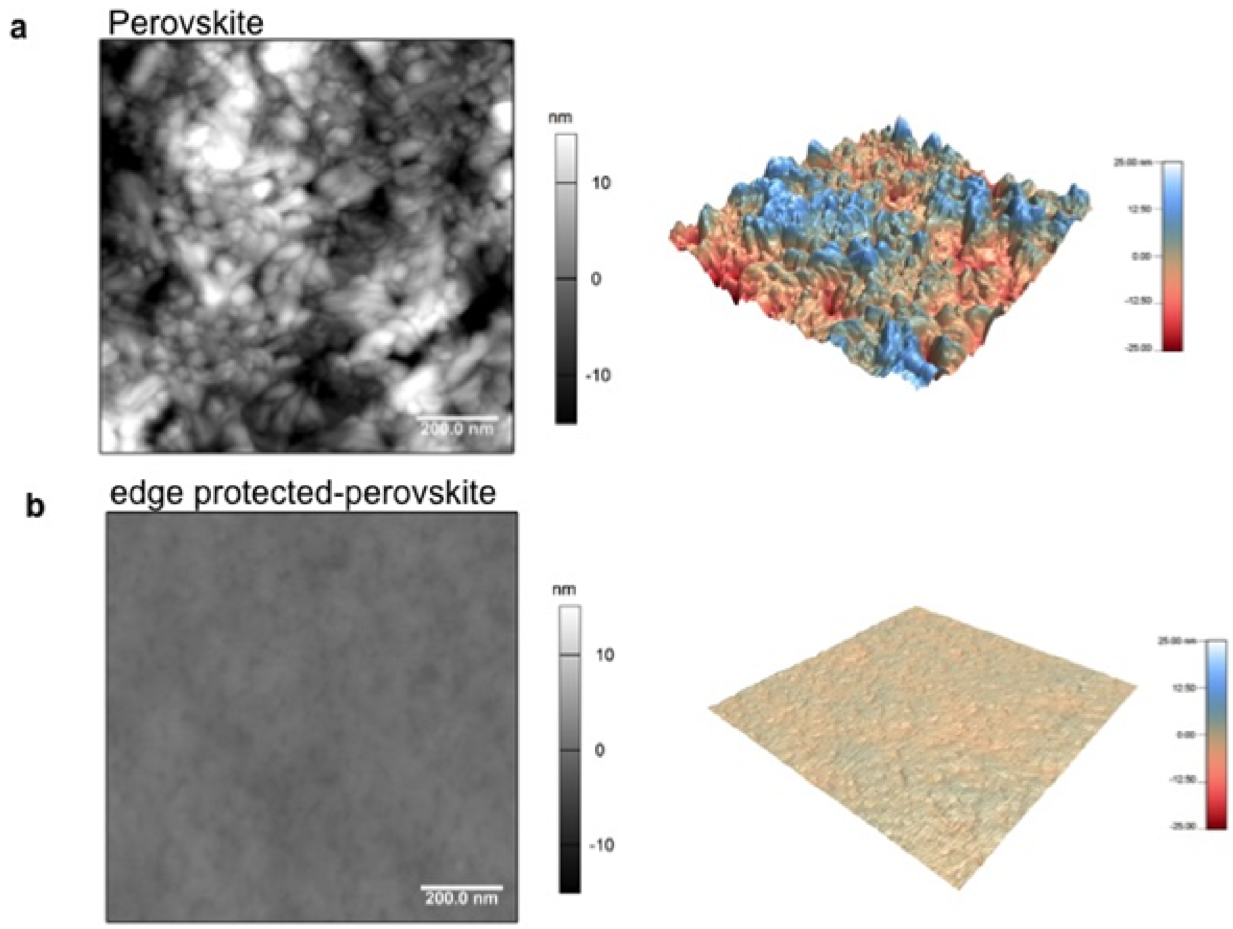

Supplementary Figure 29 | Morphology study in perovskite (a) and edge-stabilized perovskite (b) films.

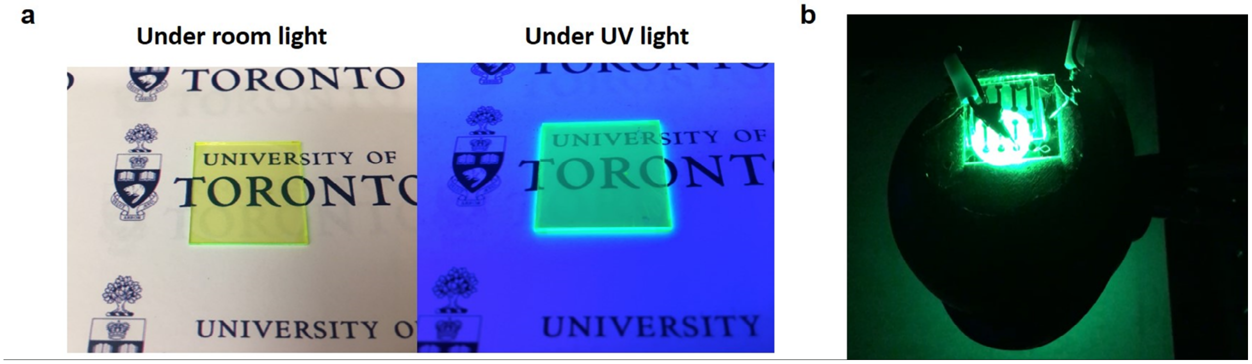

Supplementary Figure 30 | Photographs of edge-stabilized perovskite films under room light and 352nm UV light (a) and edge-stabilized perovskite LEDs (b).

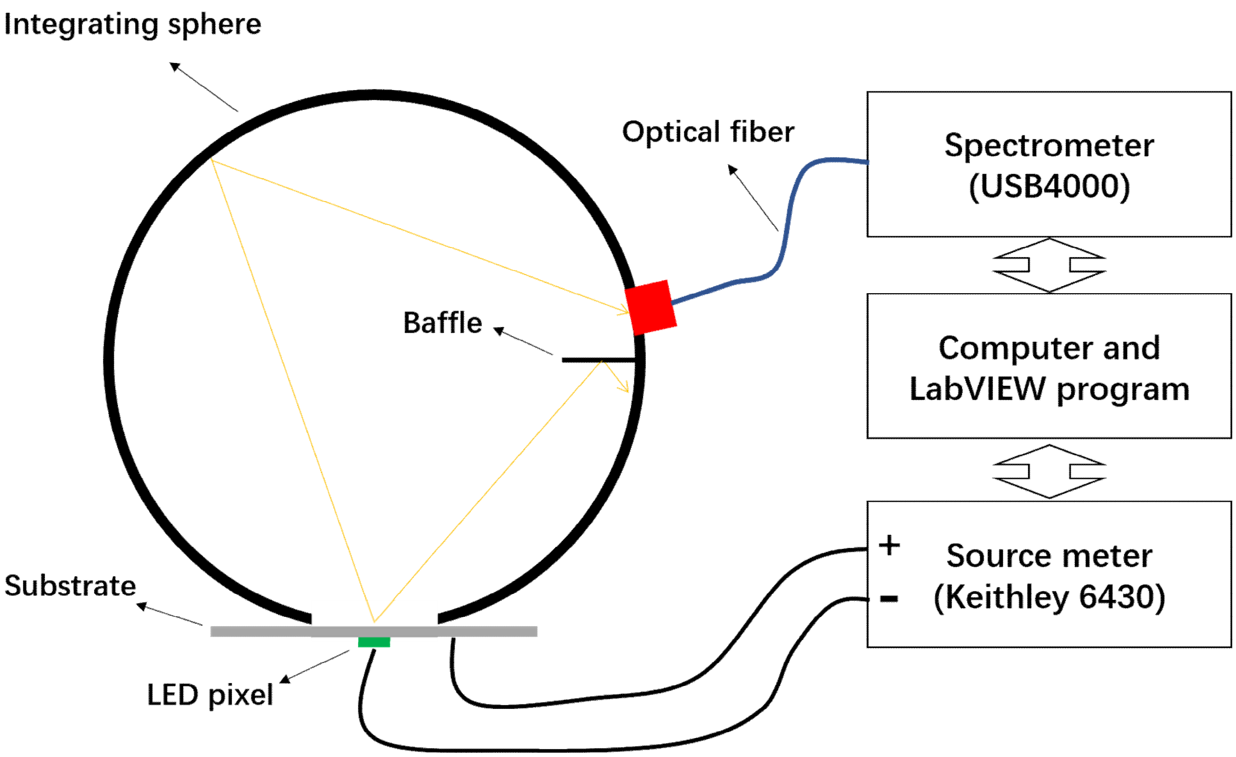

Supplementary Figure 31 | Schematic diagram of device characterization with integration sphere.

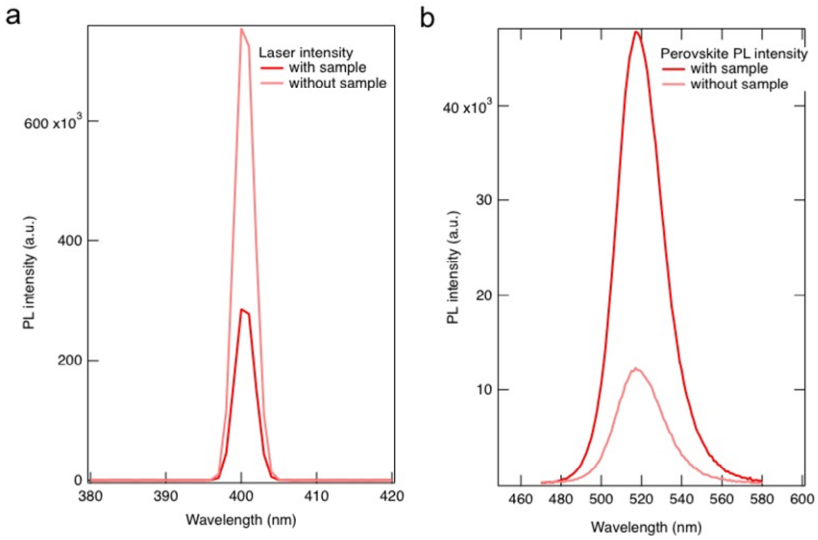

Supplementary Figure 32 | Raw spectra for obtaining absolute quantum yield. These are spectra for the edge-stabilized perovskites that exhibit near-unity quantum yield.

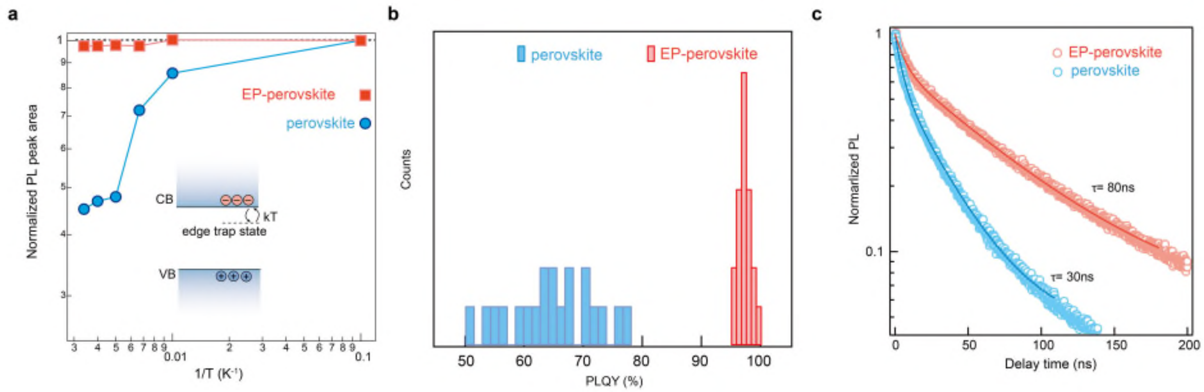

Supplementary Figure 33 | a, Temperature-dependent PL peak area of edge protected-perovskite and perovskite films. **b**, Histogram of PLQY values in perovskite and edge protected-perovskite films. **c**, Transient PL decay of perovskite and edge protected-perovskite films.

Supplementary Table 1. PLQY of different mixing ratio of Cs-MA

**Perovskites**	**PLQY (%)**
$PEA_2Cs_3Pb_4Br_{13}$	40
$PEA_2Cs_{2.4}MA_{0.6}Pb_4Br_{13}$(MA20%)	75
$PEA_2Cs_{1.5}MA_{1.5}Pb_4Br_{13}$ (MA50%)	50
$PEA_2Cs_{0.6}MA_{2.4}Pb_4Br_{13}$ (MA80%)	45
$PEA_2MA_3Pb_4Br_{13}$ (MA100%)	60

Supplementary Table 2. Summary of UPS measurement for perovskites on the different substrates

	**Substrate**	**21.2eV-Secondary cutoff (eV)**	**Offset (eV)**	**Valence band (eV)**	**ΔeV**
Perovskite	Au	5.27	0.65	5.92	0.23
Edge-stabilized perovskites	Au	5.04	0.65	5.69	
Perovskite	Si	3.08	2.95	6.03	0.3
Edge-stabilized perovskites	Si	2.45	3.28	5.73	
Perovskite	ITO	4.56	1.3	5.86	0.19
Edge-stabilized perovskites	ITO	3.94	1.73	5.67	
Perovskite	ITO-2	4.08	1.98	6.06	0.38
Edge-stabilized perovskites	ITO-2	3.58	2.1	5.68	

Supplementary Methods

Fabrication of perovskite crystals

The perovskite crystals were prepared by combining $PbBr_2$, CsBr and PEA in appropriate molar ratios in a HBr solvent mixture following a previous reported method[1].

Electron-hole only device fabrication

Electron only device was fabricated with a structure of ITO/TiO_2(50nm)/perovskite/TPBi (10nm)/LiF(1nm)/Al(100nm), and hole only device was fabricated with a structure of ITO/PEDOT:PSS:PFI/perovskite/CBP(40nm)/MoO_x(5nm)/Al(100nm) to extract the mobility and carrier injection balance.

Photoluminescence decay mapping

As synthesized perovskite crystals were mechanically exfoliated to generate micron-sized crystals, which were then dispersed into toluene (spectroscopy grade, Sigma-Aldrich). The resulted suspension was drop-casted onto a microscopy glass cover slide. Right after the sample was dried, it was submitted for photoluminescence (PL) measurements. These polycrystals have a preferred stacking orientation along the <001> direction, however still contain plently of nanoplatelet edges or grain boundaries inside the macroscopic volume. PL decay imaging was carried out on a confocal microscope (TCS-SP8, Leica). A pulsed laser emitting at 405 nm was used for excitation. The repetition rate was 5 MHz. An oil-immersion objective (magnification 20X, NA=0.75, Leica) was used for measurements. A set of time-correlated single-photon counting (TCSPC) system (PicoQuant) with an avalanche photodiode (APD) was used for time-resolved emission photon detection. The laser beam was scanned across the imaging area, with a pixel size of 280 nm and a dwell time of 1 s per pixel. PL spectral mapping was carried on a confocal microscope (Olympus). The 488 nm line from a continuous-wave argon laser was used as the excitation source. An air objective (60X, NA=0.92) was used. The emission light was collected by the same objective and was sent to a spectrometer (Horiba).

AFM measurements

Sample morphology was characterized by atomic force microscopy (AFM). Topographical and phase images were obtained with an Asylum Research Cypher AFM operated in AC mode in air.

Imaging was done using ASYELEC-02 silicon probes with titanium-iridium coatings from Asylum Research. The probes had a typical spring constant of 42 N/m. Smoothness and morphology of perovskite films were measured by atomic force microscopy (AFM). Edge-stabilized perovskite films shows a smaller grain size (<50nm) and lower root mean square (r.m.s) roughness (~2nm) than the perovskite film (r.m.s ~10nm) (**Supplementary Fig. 29**).

XRD measurements

XRD measurements of the oriented films were conducted using a Rigaku MiniFlex 600 diffractometer (Bragg-Brentano geometry) equipped with a NaI scintillation counter detector and a monochromatized Cu Kα radiation source (λ = 1.5406 Å) operating at a voltage of 40kV and current of 15mA. The diffraction patterns of $PEA_2Cs_{2.4}MA_{0.6}Pb_4Br_{13}$ perovskite film revealed distinctive to compare with 3D $CsPbBr_3$ and 2D PEA_2PbBr_4 perovskites, due to the composition of different *n* grains with a preferential orientation along the substrate. We observed two additional XRD peaks in these films, at 2θ=10.11° and 20.22°, which correspond to the diffraction from $(TPPO)_2PbBr_2$ complexes[2] (**Supplementary Fig. 17 for the XRD of TPPO-precursor reference**).

XPS measurements

XPS measurements were carried out with the Thermo Scientific K-Alpha XPS system. An Al Kα source with a 400µm spot size was used for measurements to detect photo-electrons at specific energy ranges to determine the presence of specific elements.

FTIR measurements

The Thermo Scientific Nicolet iS50 ATR-FTIR was used to obtain the FTIR spectra. The prepared films were placed on top of the ATR crystal. Spectra were obtained using 16 scans with a resolution of $4cm^{-1}$, and the collection range was between 550 cm^{-1} to 4000 cm^{-1}.

Raman spectroscopy measurements

Raman spectra of TPPO, $PbBr_2$, TPPO-$PbBr_2$ (named as TPPO-precursor), perovskite, and TPPO-perovskite deposited on glass slides were collected using a 561-nm continuous-wave diode laser (Cobolt). Scattered light was collected by an aspheric collection lens in a back scattering geometry. The Rayleigh line was attenuated by a 561-nm notch filter (Semrock, Stopline). A single monochromator with a 1200 gr/mm diffraction grating (Princeton Instruments, TriVista used as single) dispersed the back-scattered light onto a CCD camera (Princeton Instruments, Pixis 400). The entrance slit-width was kept at 100-microns. A total of 10 spectra were averaged for each sample where each spectrum was collected using a 10 second exposure time for 10 accumulations (i.e. each spectrum represents a 1000-s average). The power was maintained at 80-mW. Glass background spectra were obtained by advancing the focal plane from the slides front face into its center where only glass signal was observable. A small baseline was then drawn to correct for the residual from the glass subtraction. Spectra were collected within three Raman windows to capture low, mid, and high-frequency regions. Spectra were smoothed using a 4-point binomial smoothing algorithm. The Raman shift axis for each of these regions was calibrated to a cyclohexane standard, establishing peak frequency accuracy to +/- 5-cm^{-1} (based on variation within and between windows of known peak positions).

Raman spectra of TPPO and TPPO mixed with MABr were collected using a 785 nm continuous-wave diode laser (Renishaw) and an upright inVia Raman Microscope setup (Renishaw). A 50x objective was used to focus and collect the light to and from the sample, respectively. The Rayleigh line was attenuated with an edge filter set. A 1200 gr/mm diffraction grating was used to disperse the light onto a CCD. The power was kept at 200-mW. Spectra were

taken, using a 10 second exposure time, for several Raman windows, and a baseline was subtracted from the spectra.

The TPPO Raman spectrum changes significantly upon addition to the $PbBr_2$ precursor or to the perovskite (**Supplementary Fig. 14 and 15**). The most notable difference between the TPPO and TPPO-precursor spectra are the intensities of the 997, 1003, 1030, 1156, and 1165 cm^{-1} peaks visible in the mid-frequency spectrum. The relative intensities of the features at 997 and 1003 cm^{-1} change, while the 1030, 1156 and 1165 cm^{-1} peaks are reduced in intensity. **Supplementary Fig. 14b** contains the low-frequency window spectra of TPPO and TPPO-precursor. To further confirm that the intensity change indicates the complex formed via TPPO-Pb, we also measured Raman spectrum with a mixture of TPPO and MABr (**Supplementary Fig. 15**) using a 785 nm Raman system. Since MABr contains no Pb, we expect that they should not form a complex. The Raman spectra show no intensity change compared to TPPO only, suggesting no complexation.

UPS measurements

UPS spectra of the perovskite films were measured on Au coated substrate. Photoelectron spectroscopy was performed in a PHI5500 Multi-Technique system using non-monochromatized He-Iα radiation (UPS) (hv=21.22ev). All work function and valence-band measurement were performed at a takeoff angle of 88º, with chamber pressure near 10^{-9} Torr.

Superoxide probe test

To characterize the possibility of superoxide generation from the reaction of photogenerated electrons with oxygen, we measured fluorescence emission of a hydroethidine (HE) dye solution where the perovskite film located in probe solution, exposed to UV light and blow dry air during the illumination. The rate of increase of dye emission at 610 nm is faster in perovskite, while in

edge-stabilized perovskite the process is suppressed (**Supplementary Fig. 9**) indicating that edge-stabilized perovskite suppresses superoxide generation under the continuous UV illumination. Superoxide generation was measured following the method reported elsewhere[3]. To study the role of TPPO as an inhibitor of perovskite oxidation, we added it into toluene (1M) and measured superoxide generation.

Supplementary References

1. Stoumpos C. C., Cao D. H., Clark D. J., Young J., Rondinelli J. M., Jang J. I., Hupp J. T., Kanatzidis M. G. Ruddlesden–Popper hybrid lead iodide perovskite 2D homologous semiconductors. *Chem. Mat.* **28**, 2852-2867 (2016).

2. De La Cruz C., Sheppard N. A structure-based analysis of the vibrational spectra of nitrosyl ligands in transition-metal coordination complexes and clusters. *Spectrochim Acta A Mol Biomol Spectrosc* **78**, 7-28 (2011).

3. Gomes A., Fernandes E., Lima J. L. F. C. Fluorescence probes used for detection of reactive oxygen species. *J. Biochem. Bioph. Methods* **65**, 45-80 (2005).
